# Aldehyde dehydrogenase activity is necessary for beta cell development and functionality in mice

**DOI:** 10.1007/s00125-015-3784-4

**Published:** 2015-10-31

**Authors:** Vivian Anastasiou, Elpiniki Ninou, Dimitra Alexopoulou, Julia Stertmann, Andreas Müller, Andreas Dahl, Michele Solimena, Stephan Speier, Ioannis Serafimidis, Anthony Gavalas

**Affiliations:** Paul Langerhans Institute Dresden of Helmholtz Center Munich at the University Clinic Carl Gustav Carus of TU Dresden, Fetscherstrasse 74, 01307 Dresden, Germany; DZD – German Centre for Diabetes Research, Germany, http://www.dzd-ev.de/en; Developmental Biology Laboratory, Biomedical Research Foundation of the Academy of Athens, Soranou Ephessiou 4, Athens, 11527 Greece; Deep Sequencing Group SFB655, BIOTEChnology Center (BioZ), TU Dresden, Dresden, Germany; DFG-Center for Regenerative Therapies Dresden (CRTD), Faculty of Medicine, TU Dresden, Dresden, Germany

**Keywords:** Aldehyde dehydrogenase, Beta cell development, Beta cell dysfunction, Beta cell transcriptome, Diabetes risk factor, Hyperglycaemia, Insulin secretion

## Abstract

**Aims/hypothesis:**

Pancreatic beta cells maintain glucose homeostasis and beta cell dysfunction is a major risk factor in developing diabetes. Therefore, understanding the developmental regulatory networks that define a fully functional beta cell is important for elucidating the genetic origins of the disease. Aldehyde dehydrogenase activity has been associated with stem/progenitor cells and we have previously shown that *Aldh1b1* is specifically expressed in pancreas progenitor pools. Here we address the hypothesis that *Aldh1b1* may regulate the timing of the appearance and eventual functionality of beta cells.

**Methods:**

We generated an *Aldh1b1*-knockout mouse line (*Aldh1b1*^tm1lacZ^) and used this to study pancreatic development, beta cell functionality and glucose homeostasis in the absence of *Aldh1b1* function.

**Results:**

Differentiation in the developing pancreas of *Aldh1b1*^*tm1lacZ*^ null mice was accelerated. Transcriptome analyses of newborn and adult islets showed misregulation of key beta cell transcription factors and genes crucial for beta cell function. Functional analyses showed that glucose-stimulated insulin secretion was severely compromised in islets isolated from null mice. Several key features of beta cell functionality were affected, including control of oxidative stress, glucose sensing, stimulus-coupling secretion and secretory granule biogenesis. As a result of beta cell dysfunction, homozygous mice developed glucose intolerance and age-dependent hyperglycaemia.

**Conclusions/interpretation:**

These findings show that *Aldh1b1* influences the timing of the transition from the pancreas endocrine progenitor to the committed beta cell and demonstrate that changes in the timing of this transition lead to beta cell dysfunction and thus constitute a diabetes risk factor later in life.

*Gene Expression Omnibus (GEO) accession*: GSE58025

**Electronic supplementary material:**

The online version of this article (doi:10.1007/s00125-015-3784-4) contains peer-reviewed but unedited supplementary material, which is available to authorised users.

## Introduction

Extensive genome-wide association studies have identified over 80 genomic susceptibility loci for type 2 diabetes. The majority of the discovered risk variants have been linked to beta cell dysfunction rather than to insulin resistance [1]. Thus, elucidating the developmental regulatory networks defining a fully functional beta cell is important for understanding the genetic origins of the disease and deriving mature beta cells from stem cells.

All pancreatic cell types are derived from progenitors that emerge at the posterior foregut region of the definitive endoderm and expand to form a branched epithelium surrounded by mesenchyme [2]. Subsequently, acinar progenitors are confined at the tips, while trunk cells become endocrine/duct bipotent progenitors. Neurogenin 3-positive (NGN3^+^) endocrine progenitors arise in the trunk and migrate into the mesenchyme leaving behind ductal progenitors [3]. The emergence of progenitor pools and differentiated cell types is temporally regulated but the mechanisms implicated are unknown [3, 4]. After birth, normal beta cell development continues with the maturation of the stimulus–secretion coupling machinery, the enhancement of glucose sensing, the increase in the number of insulin-containing secretory granules (SGs) and the establishment of the appropriate beta cell mass through extensive proliferation. Several transcription factors implicated in progenitor and endocrine specification also play key overlapping roles in the postnatal expansion, maturation and maintenance of adult beta cells. Their expression levels are also important for beta cell functionality [5–12]. Disturbances of the intrauterine milieu can lead to islet defects and diabetes later in life but little is known about the molecular mediators of this effect [13–15].

Aldehyde dehydrogenase (ALDH) activity is increasingly associated with stem/progenitor cells and it is hypothesised that it contributes to the maintenance of the progenitor status [16–19]. Mitochondrial *Aldh1b1* is specifically expressed in all pancreas progenitors in the mouse embryo but not in mature endocrine cells. Its expression in the adult is confined to rare centroacinar-like cells with pancreas stem/progenitor characteristics [18, 20]. Here we addressed the hypothesis that *Aldh1b1* may regulate the timing of the appearance and eventual functionality of beta cells.

## Methods

### *Aldh1b1*^tm1lacz^-knock-in mouse strain

**Fig. 1:**
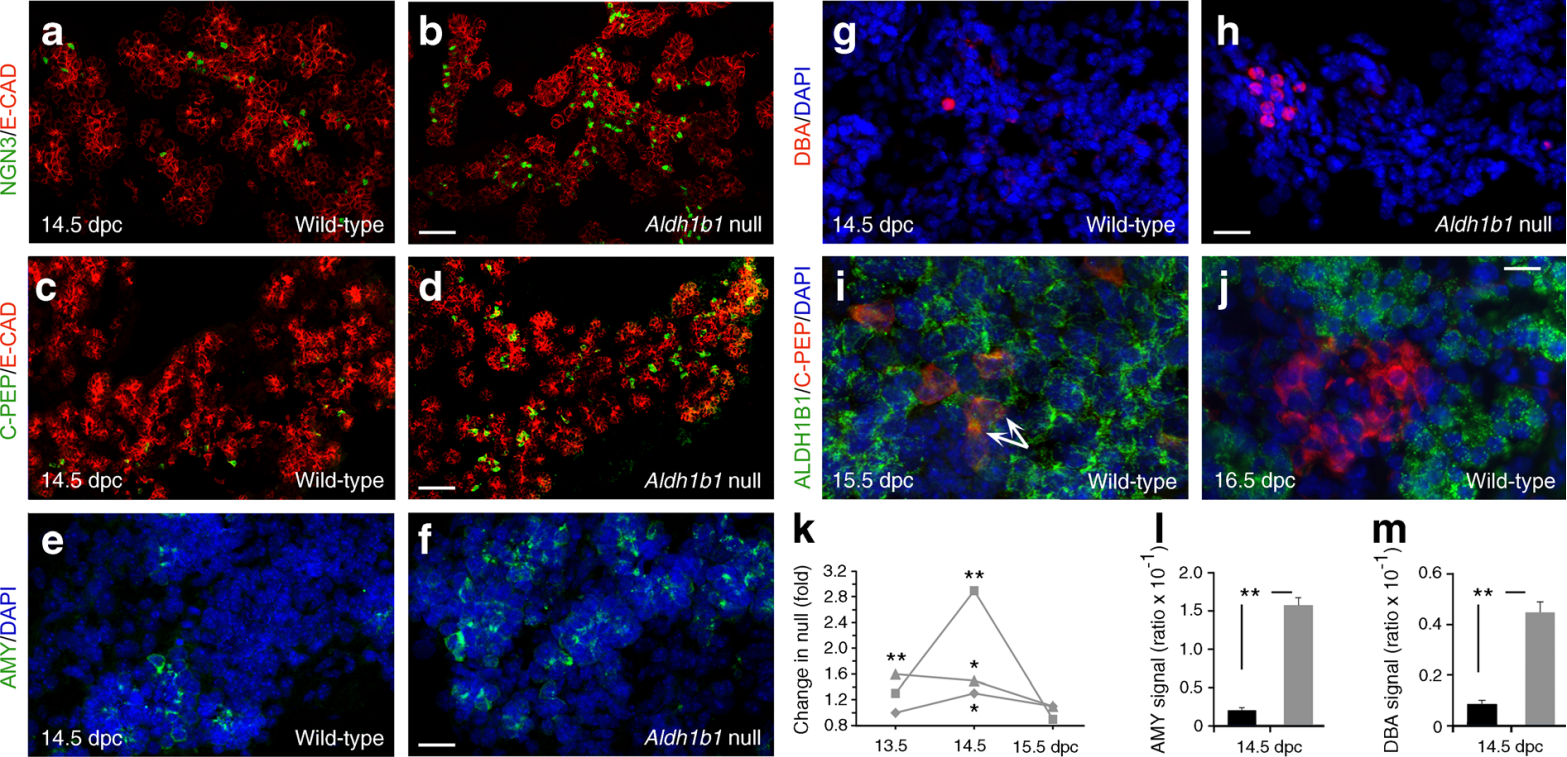
ALDH1B1 controls timing of commitment and proliferation of embryonic pancreas progenitors and is expressed in nascent beta cells. (**a**–**h**) The number of NGN3^+^ (**a**, **b**), C-PEP^+^ (**c**, **d**), AMY^+^ (**e**, **f**) and DBA^+^ (**g**, **h**) cells was increased in pancreases of *Aldh1b1* ^tm1lacZ^ null mice compared with wild-type mice. (**i**, **j**) Co-expression of ALDH1B1 with newly formed beta cells remains until 15.5 dpc (arrows) but is lost at 16.5 dpc. (**k**) Quantification of C-PEP^+^ (squares), NGN3^+^ (triangles) signal (DAPI normalised) and number of PH3^+^ epithelial (diamonds) cells per islet at 13.5, 14.5 and 15.5. dpc showed a transient increase (expressed as fold increase compared with wild-type) in *Aldh1b1* ^tm1lacZ^ null pancreases. Comparisons were done for the same day. (**l**, **m**) Quantification of AMY^+^ (**l**) and DBA^+^ (**m**) signal (DAPI normalised) at 14.5 dpc showed a strong increase (expressed as ratio over wild-type) in the *Aldh1b1* ^tm1lacZ^ null pancreases (black bars, wild-type; grey bars, null). Results are from four to six embryos per genotype and per embryonic stage, and values are representative of eight sections per embryonic pancreas spanning the entire organ. *p < 0.05 and **p < 0.01. Scale bars, 50 μm (**a**, **b**), 100 μm (**c**, **d**), 20 μm (**e**–**h**) and 15 μm (**i, j**). E-CAD, E-cadherin

The *Aldh1b1*^tm1(KOMP)Vlcg^ ES line (KOMP; UC Davis, CA, USA) was used to generate the *Aldh1b1*^tm1lacZ^ allele (electronic supplementary material [ESM] Fig. 1). Animal maintenance and experimentation were in accordance with international guidelines and subjected to ethics approval from the competent veterinary committees of Athens and TU Dresden. Animals were assigned to experiments randomly on condition of fulfilling the conditions of the experiment (genotype, sex, age) as described, and the scoring/outcome assessment was blind to group assignment.

### Fasting glucose measurements and glucose, insulin and pyruvate tolerance tests

For the intraperitoneal glucose tolerance test (IPGTT), male mice were fasted for 16 h overnight and injected intraperitoneally with d-glucose (Sigma-Aldrich, St Louis, MO, USA) at 2 g/kg. Blood from the tail vein was used to determined glucose level using the Contour XT monitoring system (Bayer, Leverkusen, Germany) and insulin level was determined in the supernatant fraction following clotting using an ultrasensitive mouse insulin ELISA kit (Mercodia, Uppsala, Sweden). Details of the intraperitoneal insulin tolerance test (IPITT) and intraperitoneal pyruvate tolerance test (IPPTT) procedures are provided in ESM Methods.

### Immunostaining and morphometric analysis

Dissected pancreases were fixed in 4% wt/vol. paraformaldehyde and processed for immunofluorescence using standard procedures [18, 21]. Details of antibodies used and morphometric analyses are provided in ESM Methods.

### Islet isolation and functional analyses

Islets were isolated from male mice and maintained as described [22]. Standard procedures were used for insulin secretion assays, measurements of intracellular calcium ([Ca^2+^]_i_) changes [23], detection of reactive oxygen species (ROS) [24], ATP measurements [25] and transmission electron microscopy [26]. Details are provided in ESM Methods.

### RNA isolation, real-time PCR, RNA Seq and accession numbers

Total RNA was prepared using the RNeasy kit (Qiagen, Hilden, Germany), first-strand cDNA preparation, real-time PCR and library preparations for RNA sequencing and bioinformatics analyses were done according to standard procedures. Details are provided in ESM Methods. Raw and normalised data were deposited in GEO (www.ncbi.nlm.nih.gov/geo) under accession number GSE58025.

### Hormone measurements and liver glycogen content assay

Pancreatic insulin, proinsulin and glucagon content, as well as liver glycogen content, were assayed as described in ESM Methods.

### Western blotting and X-gal staining

Details on western blotting and X-gal staining procedures are provided in ESM Methods.

### Statistical analyses

Statistical significance was determined by Student’s *t* test for two-tailed distributions of unpaired groups. For analyses of wild-type and null mice at different time points (with the exception of gestation time points) two-way ANOVA with Bonferroni post hoc test for differences between means were conducted. The SEM is provided unless otherwise stated and *p* < 0.05 was considered significant. For differences in islet size distributions and means, statistical significance was determined using the Wilcoxon rank-sum test and *p* < 0.001 was considered significant.

## Results

### Premature lineage commitment during development in the absence of *Aldh1b1* expression

We generated the *Aldh1b1*^tm1lacZ^ mouse line (ESM Fig. 1 a, b) and analysed pancreas development in the *Aldh1b1*^tm1lacZ^ null embryos in which *Lacz* expression recapitulated expression in the pancreas (ESM Fig. 1 c–h).

Early development appeared normal (data not shown) but progenitor differentiation was accelerated during the secondary transition. There was a transient increase in the number of NGN3^+^ cells at 13.5 and 14.5 days post coitum (dpc) (Fig. 1a, b, k) and, consistently, the number of C-peptide-positive (C-PEP^+^) cells (but not glucagon-positive [GCG^+^] cells, data not shown) was increased in the *Aldh1b1*^tm1lacZ^ null pancreases (Fig. 1c, d, k). Amylase complex-positive (AMY^+^) acinar cells and duct Dolichos biflorus agglutinin-positive (DBA^+^) cells appeared earlier and were more numerous in the *Aldh1b1*^tm1lacZ^ null pancreases at 14.5 dpc (Fig. 1e–h, l, m).

The number of epithelial cells in mitosis (Phosphohistone-H3^+^/E-cadherin^+^ cells) transiently increased in the *Aldh1b1*^tm1lacZ^ null pancreases at 14.5 dpc, thus compensating for the accelerated differentiation and maintaining the size of the organ (Fig. 1k and data not shown). Apoptosis levels, detected by immunofluorescence for activated caspase-3, were very low and similar to controls excluding implication of ALDH1B1 in progenitor survival (data not shown).

Expression of the sex determining region Y-box 9 (SOX9), pancreatic and duodenal homeobox 1 (PDX1) and pancreas specific transcription factor 1a (PTF1a) progenitor markers at 14.5 dpc and RNA Seq analysis of 13.5 and 15.5 dpc total pancreases did not detect other significant quantifiable differences between wild-type and *Aldh1b1*^tm1lacZ^ null pancreases, suggesting that only a subset of progenitor cells are affected at successive time points. Careful analysis of ALDH1B1 expression showed that the protein persisted in nascent C-PEP^+^ cells until 15.5 dpc but not later (Fig. 1i, j and data not shown).

Therefore, loss of *Aldh1b1* function resulted in premature differentiation, balanced by increased mitosis, suggesting a dual role for *Aldh1b1* in regulating both timing of commitment and proliferation of progenitors. We then asked whether loss of ALDH1B1 in progenitors and nascent beta cells affected islet patterning and beta cell function.

### Islet patterning is defective in newborn *Aldh1b1*^tm1lacZ^ null mice

Cellular analysis revealed a striking heterogeneity of *Aldh1b1*^tm1lacZ^ null islets at postnatal day 1 (P1). Expression of the transcription factors PDX1 and NK6 homeobox 1 (NKX6.1) [12, 27–30] was homogeneous in control islets whereas a large number of *Aldh1b1*^tm1lacZ^ null islets showed absent, substantially weaker NKX6.1 expression (47%) or strongly increased expression (18%). Some (35%) showed substantially weaker PDX1 expression compared with controls and co-expression of NKX6.1 and PDX1 within cells of the same islet was not uniform (ESM Fig. 2 a, b, b’ and data not shown) (*n* = 76). This was accompanied by variable levels of C-PEP immunofluorescence in the *Aldh1b1*^tm1lacZ^ null P1 pancreases, where 50% of the islets had either reduced or absent staining compared with controls (ESM Fig. 2c, d, d’). In addition, the number of phosphohistone-H3 (PH3)^+^ cells was increased by nearly 40% in the null islets (ESM Fig. 2f and data not shown). However, *Aldh1b1*^tm1lacZ^ P1 null pancreases had similar weight, beta cell mass, islet size distribution and insulin, proinsulin and glucagon content compared with controls. The relative numbers of alpha, beta and delta cells remained similar (Fig. 4d, ESM Figs 2 e, 3 g–j, l and data not shown).

**Fig. 2:**
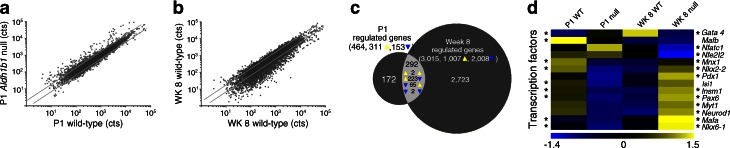
Islet transcriptome analysis at P1 and week 8 (WK 8). (**a**, **b**) Scatter plots of normalised gene transcription counts (cts) of *Aldh1b1* ^tm1lacZ^ null islets vs wild-type (WT) islets at P1 (**a**) (Pearson’s *r* = 0.63) and WK 8 (**b**) (Pearson’s *r* = 0.56). Diagonal lines represent regulation cut-offs at 0.6- and 1.6-fold. (**c**) Proportional Venn diagram of the genes misregulated in *Aldh1b1* ^tm1lacZ^ null islets at P1, WK 8 or both (overlap). In the overlap, the direction of regulation at P1 (left) or WK 8 (right) is shown for genes regulated at both time points. (**d**) Heat map of relative expression values (*z* scores) of transcription factors in *Aldh1b1* ^tm1lacZ^ null and control pancreases at P1 and WK 8. *p_adj_ ≤ 0.1: at the left side of the heat map * refers to comparisons at P1 whereas at the right side it refers to comparisons at WK 8

Western blots and RNA Seq data confirmed that *Aldh1b1* is not expressed in the islets at postnatal and adult stages [18] (ESM Fig. 2g). Transcriptome comparison of P1 *Aldh1b1*^tm1lacZ^ null and control islets showed deregulation of a substantial number of genes (Fig. 2a, c). This deregulation included repression of several transcription factors with key roles in beta cell maturation and maintenance, such as *Pdx1*, *Nkx6.1*, *Mafa* and *Mafb* (Fig. 2d and ESM Table 1), and misregulation of several genes encoding vesicular and SG proteins (see below).

Taken together these data suggest that *Aldh1b1* is necessary for proper fetal development of beta cells and the upregulation of key transcription factors.

### Islet patterning defects are maintained and expanded in adulthood

**Fig. 3:**
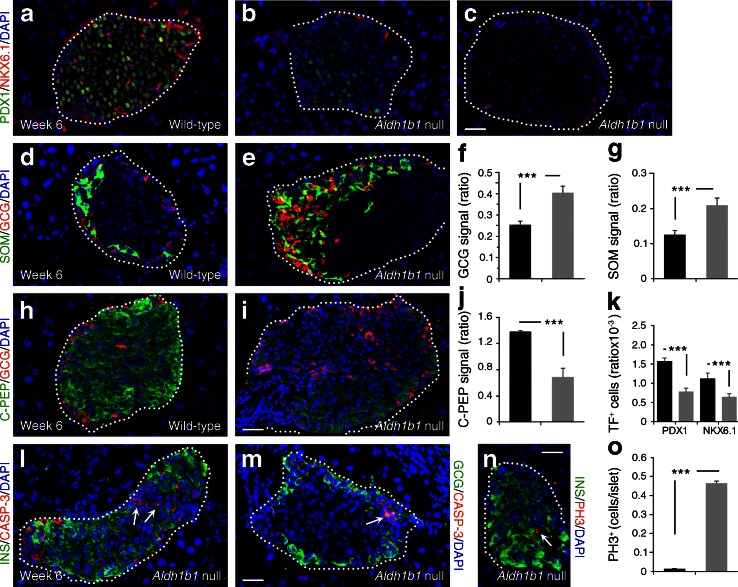
Islet patterning defects in *Aldh1b1* ^tm1lacZ^ null young adult mouse islets. (**a**–**c**, **h**–**i**) Substantially lower (**b**) or absent (**c**) PDX1, NKX6.1 (**a**–**c**) and C-PEP (**h**, **i**) expression in *Aldh1b1* ^tm1lacZ^ null islets. (**d**–**g**) Quantitative analysis of glucagon (GCG) and SOM (**d**, **e**) signal per islet (expressed as ratio of signal over DAPI signal) shows that the signal is increased by 60 and 65%, respectively (**f**, **g**), in *Aldh1b1* ^tm1lacZ^ null islets (black bars, wild-type; grey bars, null). (**j**, **k**) Quantitative analysis of C-PEP signal (**j**) and PDX1^+^, NKX6.1^+^ cells (**k**) per islet (expressed as ratio of signal over DAPI signal) shows that their expression is significantly reduced in *Aldh1b1* ^tm1lacZ^ null islets (black bars, wild-type; grey bars, null). (**l**, **m**) *Aldh1b1* ^tm1lacZ^ null islets showed increased levels of apoptosis as indicated by double immunofluorescence for caspase-3 (CASP-3) and either insulin (INS) (**l**) or GCG (**m**) (arrows). (**n**, **o**) Immunofluorescence for PH3 (**n**) indicated that mitotic activity was 50-fold higher (**o**) in *Aldh1b1* ^tm1lacZ^ null islets (black bars, wild-type; grey bars, null). Results are from three to five animals per genotype, and values are representative of scoring at least 50 islets spanning the entire pancreas of each animal. Values are mean ± SEM. ***p < 0.001 for indicated comparisons. Scale bars, 25 μm (**a**–**e**, **h**, **i**, **l**–**n**). TF, transcription factor

**Fig. 4:**
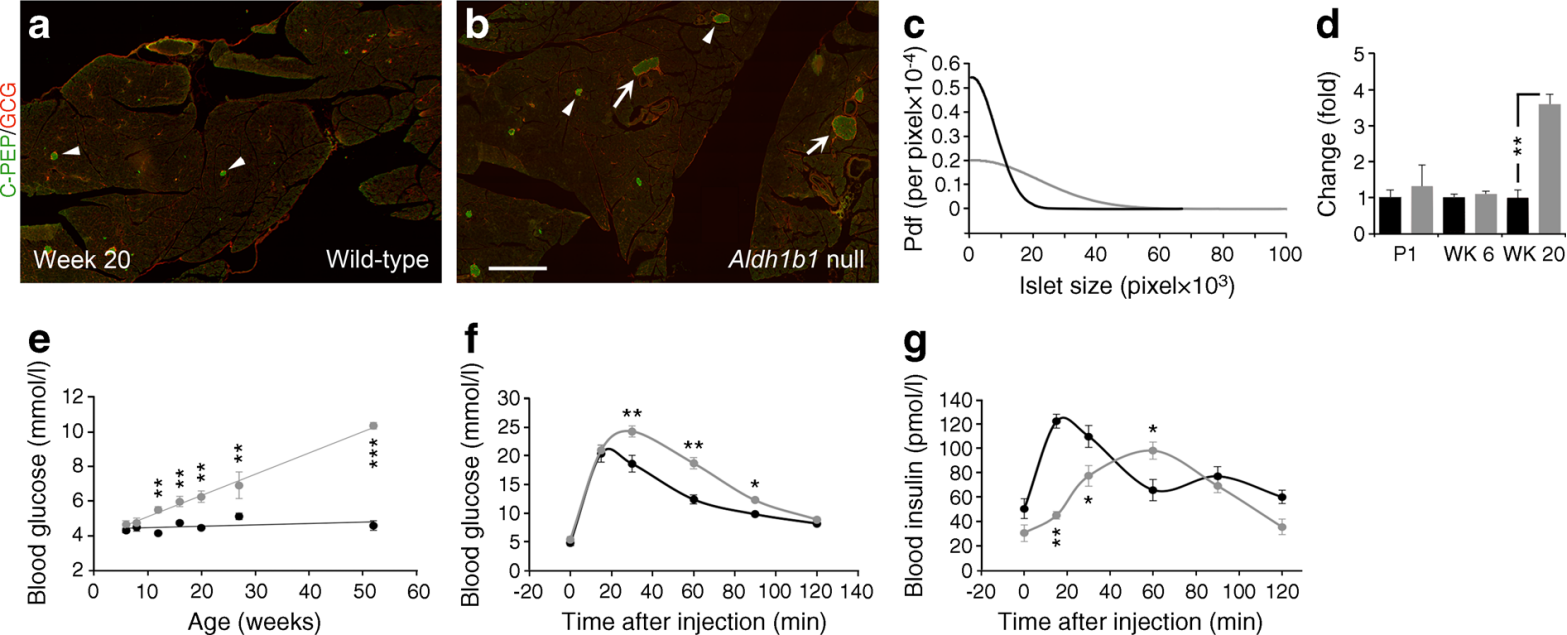
*Aldh1b1* ^tm1lacZ^ null mice have larger islets but are glucose intolerant and develop age-dependent hyperglycaemia. (**a**, **b**) Double immunofluorescence for C-PEP and glucagon (GCG) indicated the presence of both normal-sized islets (arrowheads) and significantly larger islets (arrows) in *Aldh1b1* ^tm1lacZ^ null mice compared with wild-type mice. (**c**) Morphometric analysis and plotting of the islet size probability density function (pdf) illustrates the significantly larger *Aldh1b1* ^tm1lacZ^ null median islet size (3,223) compared with the wild-type (1,413) (black line, wild-type; grey line, null) at week 20. Sizes are expressed in pixels (100 μm = 135 pixels). Differences in the distribution are significant (*p* < 0.001). (**d**) Beta cell mass differences are negligible at P1 and week 6 (WK 6) but there is a 3.6-fold increase in the beta cell mass of the *Aldh1b1* ^tm1lacZ^ compared with that of wild-type at week 20 (WK 20) (black bars, wild-type; grey bars, null). **p < 0.01 for the indicated comparison. (**e**) Blood glucose levels after overnight fasting in 6- to 52-week-old mice showed that *Aldh1b1* ^tm1lacZ^ nulls gradually develop hyperglycaemia (black line, wild-type; grey line, null) (*n* = 8–20). (**f**) *Aldh1b1* ^tm1lacZ^ nulls show a significant delay in blood glucose clearance during IPGTT at week 8 (*n* = 8, 7) (black line, wild-type; grey line, null). (**g**) Acute insulin secretion during IPGTT is impaired at WK 8 in *Aldh1b1* ^tm1lacZ^ null mice compared with wild-type mice (*n* = 5) (black line, wild-type; grey line, null). Results in (**a**–**d**) are from three animals per genotype and per age group, and values are representative of 25 sections spanning the entire pancreas of each animal. Results in (**e**–**g**) are from the indicated number of animals (*n*) per genotype. Values are mean ± SEM. *p < 0.05, **p < 0.01 and ***p < 0.001, *Aldh1b1* ^tm1lacZ^ null vs wild-type. Scale bar, 1.5 mm

The murine pancreas undergoes dramatic remodelling during the postnatal period when beta cell mass expands and beta cells acquire the capacity to secrete insulin in response to glucose [31, 32]. Heterogeneity in *Aldh1b1*^tm1lacZ^ null islets persisted in early adults at 6 weeks of age. Nearly one-third of null islets (30%) showed substantially lower or absent expression of both PDX1 and NKX6.1, one-third (37%) consisted of a mixture of PDX1^+^ and NKX6.1^+^ cells and the remaining 33% had normal PDX1 and NKX6.1 expression (*n* = 123) (Fig. 3a–c, k). Changes in the expression of these genes have been associated with increased numbers of GCG^+^ and somatostatin (SOM)^+^ cells [12, 25]. Accordingly, many null islets contained supernumerary GCG^+^ and SOM^+^ cells and often had disrupted architecture (Fig. 3d–g). There was no apparent interconversion among endocrine cells since we were unable to detect any C-PEP^+^/GCG^+^ or insulin-positive (INS)^+^/SOM^+^ cells (data not shown). Furthermore, 50% of the islets, particularly larger ones, had low or even absent C-PEP and INS immunoreactivity while maintaining normal GLUT2 and glucagon-like peptide-1 receptor (GLP1R) expression (Fig. 3h–j and ESM Fig. 4 a–d). Null islets showed ectopic caspase-3 immunoreactivity in both normally and weakly C-PEP^+^ null islets (Fig. 3l, m) and persistent mitotic activity, mostly in those exhibiting low C-PEP staining (Fig. 3n, o), thus maintaining beta cell mass and median islet size (Fig. 4d and ESM Fig. 3a). Heterogeneity persisted at week 20, particularly concerning low C-PEP and NKX6.1 immunofluorescence, mostly in larger islets (ESM Fig. 4 e–h).

These findings showed that defects and heterogeneity in islet patterning persisted into adulthood and to determine their extent we compared the transcriptome of *Aldh1b1*^tm1lacZ^ null and control islets isolated at week 8. The number of misregulated genes had now expanded more than sixfold (Fig. 2b, c) and gene ontology analyses suggested that key components of beta cell function were affected (see below). Strikingly, expression of most transcription factors necessary to maintain islet functionality, with the notable exception of the downregulation of *Nfe2l2* (*Nrf2*), a gene protecting beta cells from oxidative stress [33], was upregulated, suggesting an effort towards functional compensation (Fig. 2d and ESM Table 1). Consistent with the increased mitosis rates, expression of several cell cycle and mitosis-associated genes was altered in the *Aldh1b1*^tm1lacZ^ null islets (ESM Fig. 5a and ESM Table 2). Continued mitosis in the islets resulted in a 3.6-fold expansion of the beta cell mass and a twofold increase in the median islet size in *Aldh1b1*^tm1lacZ^ nulls by week 20 (Fig. 4a–d, ESM Fig. 2e and ESM Fig. 3a).

These findings showed that early defects persisted and expanded in the islets of *Aldh1b1*^tm1lacZ^ null mice, suggesting impaired functionality.

### Null mice are glucose intolerant and develop age-dependent hyperglycaemia

To establish whether these molecular defects compromised glucose homeostasis we first determined blood glucose levels following overnight fasting of 6- to 52-week-old mice. From 12 weeks onwards *Aldh1b1*^tm1lacZ^ null mice developed hyperglycaemia, which accelerated with age (Fig. 4e) [34]. IPGTTs showed that the onset of hyperglycaemia was preceded by reduced glucose tolerance and significant delay in acute insulin secretion in young adult *Aldh1b1*^tm1lacZ^ null mice. The first-phase response in the *Aldh1b1*^tm1lacZ^ null mice took nearly three times as long to peak without reaching control levels and a second-phase response was absent (Fig. 4f, g). Reduced glucose tolerance was further exacerbated at week 20 and acute insulin secretion was equally weak at week 20 (ESM Fig. 3b, c).

Hormone levels were not reduced at week 8 or week 20 in *Aldh1b1*^tm1lacZ^ null pancreases and pancreas weight was similar to that in wild-type controls (see above and data not shown). In fact, there was a transient increase in insulin and proinsulin content as well as a 2.6-fold increase in glucagon content in the null pancreases at week 8 (ESM Fig. 3j). The insulin-to-proinsulin ratio remained remarkably similar between *Aldh1b1*^tm1lacZ^ null and controls until week 20 (ESM Fig. 3g–i). Total pancreas hormone levels returned to normal at week 20 and this, coupled with the 3.6-fold expansion in beta cell mass at that stage (ESM Figs 3g, h, j and Fig. 4d), implied lower insulin concentration in cells.

ALDH1B1 is not expressed in the fat or skeletal muscle of adult mice, as demonstrated by the lack of detectable β-galactosidase activity in *Aldh1b1*^tm1lacZ^ heterozygotes (data not shown). To exclude the possibility that the defects in glucose homeostasis observed in *Aldh1b1*^tm1lacZ^ null mice are due to secondary defects arising from the liver or other peripheral tissues, we conducted a series of experiments that included IPITTs (ESM Fig. 3d), measurement of glucagon levels in the blood (ESM Fig. 3l), IPPTTs and measurement of glucose-6-phosphatase gene transcript levels as indicators of gluconeogenesis (ESM Fig. 3m, n), and measurement of hepatic glycogen stores (ESM Fig. 3m). In all assays, *Aldh1b1*^tm1lacZ^ null mice behaved similarly to wild-type controls.

Taken together, these findings suggested that the *Aldh1b1*^tm1lacZ^ null mice are glucose intolerant due to islet defects in glucose-stimulated insulin secretion (GSIS).

### *Aldh1b1*^tm1lacZ^ null islets are defective in GSIS

**Fig. 5:**
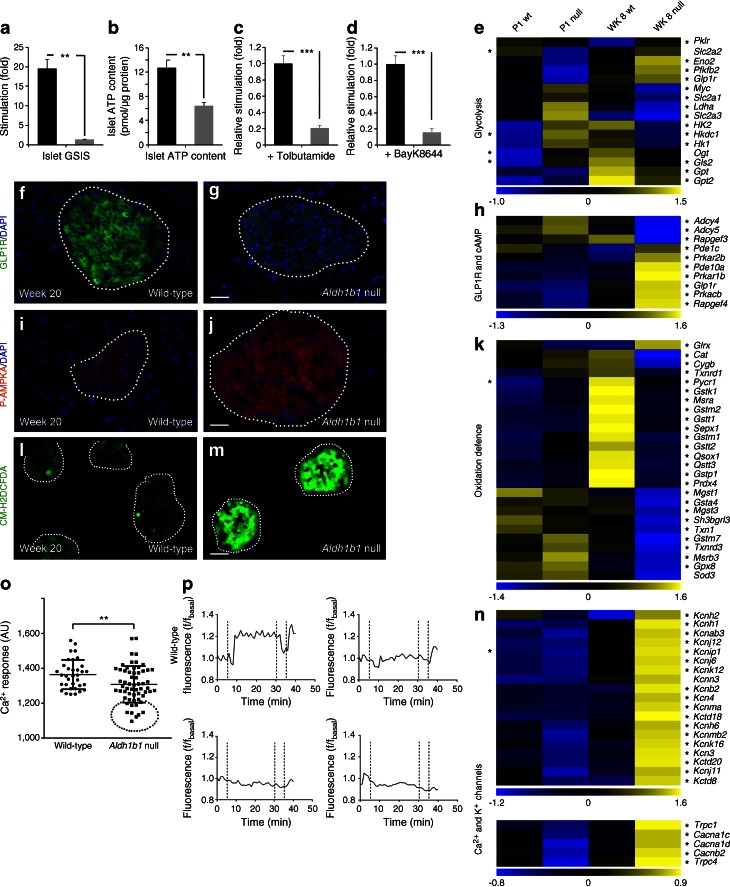
Glucose sensing and stimulus-coupling secretion are impaired in *Aldh1b1* ^tm1lacZ^ null mice. (**a**, **b**) GSIS (**a**) and total ATP content (**b**) is reduced by 13-fold and twofold, respectively, in islets isolated from *Aldh1b1* ^tm1lacZ^ null mice compared with those isolated from wild-type mice (*n* = 3) (black bars, wild-type; grey bars, null). (**c**, **d**) Stimulation of insulin release in isolated islets with tolbutamide (**c**) or BayK8644 (**d**) is reduced by fivefold in the *Aldh1b1* ^tm1lacZ^ nulls compared with those isolated from wild-type mice (*n* = 3) (black bars, wild-type; grey bars, null). (**e**–**n**) Immunofluorescence and gene expression analyses showed that *Aldh1b1* ^tm1lacZ^ null islets have dramatically reduced GLP1R expression (**f**, **g**), are energy depleted (**i**, **j**) and contain high levels of ROS (**l**, **m**). *z* score heat maps showed misregulated expression of several genes involved in glycolysis (**e**), GLP1R-mediated cAMP production (**h**) and oxidation defence (**k**), as well as Ca^2+^ and K^+^ channel genes (**n**) in the *Aldh1b1* ^tm1lacZ^ null islets. (**o**, **p**) Increase in intracellular Ca^2+^ concentration, calculated as area under the curve in arbitrary units (AU), following glucose stimulation, is reduced in *Aldh1b1* ^tm1lacZ^ null islets (**o**). A subset of *Aldh1b1* ^tm1lacZ^ null islets (circled in **o**) shows weak or absent intracellular Ca^2+^ mobilisation when compared with a typical wild-type response (wild-type response is shown in the upper left panel of **p**, the others represent affected islets). Horizontal lines in (**o**) represent the mean and SD; dotted lines in (**p**) represent injection time points of stimulation medium (5 min 20 s), baseline (35 min 20 s) and KCl (40 min 20 s). Results are from three to five animals per genotype. At least 25 islets per animal were scored or assayed. Values are means ± SEM. **p < 0.01 and ***p < 0.001 for indicated comparisons. Heat maps (**e**, **h**, **k**, **n**), *p_adj_ ≤ 0.05: at the left side of the heat map * refers to comparisons at P1 whereas at the right side it refers to comparisons at WK 8. Scale bars, 25 μm (**g**, **j**) and 80 μm (**m**). WK, week; WT, wild-type

*Aldh1b1*^tm1lacZ^ null mice are born with islet molecular defects that are expanded in early adulthood. The high insulin content in the pancreases of adult *Aldh1b1*^tm1lacZ^ null mice at week 8 (ESM Fig. 3g) partially compensated for functional defects and delayed the onset of hyperglycaemia, which became evident from week 12 and worsened progressively with age (Fig. 4e). Therefore, we focused subsequent functional analyses of the islets at week 20. Serum insulin levels were similar in *Aldh1b1*^tm1lacZ^ null and control mice, following a 4 h fasting period (ESM Fig. 3k) but following a glucose challenge the defects in acute insulin secretion of *Aldh1b1*^tm1lacZ^ null mice were similar to those detected at week 6 (Fig. 4g and ESM Fig. 3c). We then directly assayed the GSIS capacity of islets isolated from *Aldh1b1*^tm1lacZ^ null mice at week 20. Insulin medium concentration in the basal condition was 79.2 ± 8.6 pmol/l in wild-type islets and 87.8 ± 6.9 pmol/l in *Aldh1b1*^tm1lacZ^ null islets. *Aldh1b1*^tm1lacZ^ null islets responded very weakly to glucose (Fig. 5a), with insulin concentration reaching 118.8 ± 5.2 pmol/l whereas in wild-type islets it reached 1.72 ± 0.22 nmol/l.

These findings showed that *Aldh1b1*^*tm1lacz*^ null islets had severe defects in glucose sensing, stimulus-coupled insulin secretion or both.

### Glucose sensing is impaired in the *Aldh1b1*^tm1lacZ^ null islets

We then investigated glucose uptake, glycolytic flux and ATP generation in the *Aldh1b1*^tm1lacZ^ null mutants. Immunostaining for *Glut2* (*Slc2a2*) did not reveal changes in expression at either week 6 or week 20 (ESM Fig. 4 i, j). However, GLP1R immunostaining was severely decreased in all *Aldh1b1*^tm1lacZ^ islets, indicating the loss of its potentiating effect in glucose uptake (Fig. 5f, g). RNA Seq data showed that expression of *c-myc*, a central regulator of glycolytic gene expression [35], was strongly repressed and expressions of several genes involved in the glycolytic pathway and genes encoding enzymes diverting glutamine in the tricarboxylic acid cycle were deregulated (Fig. 5e and ESM Table 1). In mammals, low energy levels are reflected by increased AMP:ATP or ADP:ATP ratios and this leads to the activation of AMP-activated protein kinase (AMPK) by phosphorylation [36]. Levels of P-AMPKA in the *Aldh1b1*^tm1lacZ^ null islets were substantially higher than in controls suggesting that null beta cells were energy depleted (Fig. 5i, j). ATP levels, determined by chemiluminescence, were indeed reduced by nearly twofold in isolated *Aldh1b1*^tm1lacZ^ null islets (Fig. 5b). Additionally, a very large number of ribosomal protein genes were downregulated (ESM Fig. 5e and ESM Table 2). This also explains the weak expression of PDX1 and NKX6.1 at the protein level (Fig. 3a–c) despite the upregulation of the corresponding genes (Fig. 2d and ESM Table 1).

Beta cells are vulnerable to sustained oxidative stress due to the low expression of antioxidant enzymes [37]. Control mice maintained or induced expression of several genes involved in protection from and repair of oxidative damage in the transition from P1 to the adult stage. This induction did not take place in the *Aldh1b1*^tm1lacZ^ null islets (Fig. 5k and ESM Table 1), consistent with the repression of *Nfe2l2* (Fig. 2d) [33]. Staining of islets isolated at week 20 with the live ROS indicator CM-H2DCFDA indicated a much higher ROS content in the null islets (Fig. 5l, m). Additionally, immunofluorescence for 4-hydroxynonenal, an indicator of excessive oxidation of unsaturated fatty acids, showed that *Aldh1b1*^tm1lacZ^ null islets were more strongly stained (ESM Fig. 4k, l). Thus, these findings suggested that *Aldh1b1*^tm1lacZ^ null islets are subject to oxidative stress.

Taken together these data showed that *Aldh1b1*^tm1lacZ^ null islets are energy depleted and exposed to high oxidative load.

### Stimulus-coupling secretion is impaired in the *Aldh1b1*^tm1lacZ^ null islets

We then investigated the expression and function of the constituent components of stimulus-coupling secretion machinery. The RNA Seq data showed that expression of several K^+^ and Ca^2+^ transporters was misregulated in the *Aldh1b*^tm1lacZ^ islets (ESM Fig. 5b and ESM Table 2). Additionally, there was a strong upregulation of several K^+^ channel genes in *Aldh1b1*^tm1lacZ^ null islets, including *Kcnj11*, a type 2 diabetes risk factor [38] (Fig. 5n and ESM Table 1), and expression of adenylate cyclases *Adcy4* and *Adcy5* was repressed (Fig. 5h and ESM Table 1). To determine whether defects from membrane depolarisation and downstream of it contributed to the phenotype, we first stimulated insulin secretion in isolated islets with 100 μmol/l tolbutamide, a K^+^ channel antagonist, in the presence of basal levels of glucose. Following stimulation, the insulin concentration went from 79.2 ± 8.6 pmol/l to 13.9 ± 0.15 nmol/l in the wild-type islet medium and reached just 3.27 ± 0.15 nmol/l in the *Aldh1b1*^tm1lacZ^ null islet medium, suggesting that the efficiency of events starting with K^+^-mediated membrane depolarisation and leading to insulin secretion was compromised (Fig. 5c).

The L-type voltage-gated Ca^2+^ channel genes *Cacna1c* and *Cacna1d* are essential for rodent pancreatic beta cell function [39, 40] and polymorphisms in *Cacna1d* have been associated with type 2 diabetes [41]. RNA Seq data showed that Ca^2+^ channel genes, including *Cacna1c* and *Cacna1d*, were upregulated in the adult *Aldh1b1*^tm1lacZ^ islets (Fig. 5n and ESM Table 1). We isolated potential K^+^ channel defects by directly stimulating Ca^2+^ influx in isolated islets with 2 μmol/l of the specific L-type voltage-sensitive Ca^2+^ channel agonist BayK8644 in the presence of basal levels of glucose. Following stimulation of wild-type islets, insulin concentration in the medium reached 0.58 ± 0.04 nmol/l but remained virtually unchanged in the *Aldh1b1*^tm1lacZ^ null islets, suggesting that increased expression of voltage-gated Ca^2+^ channels was not reflected at the protein level and/or that formation of SGs and exocytosis were affected (Fig. 5d). We then followed, by fluorescence, changes in the intracellular Ca^2+^ concentration in response to glucose stimulation in isolated islets. The average response was reduced in the week 20 *Aldh1b1*^tm1lacZ^ null islets (six mice, *n* = 59) compared with the wild-type controls (four mice, *n* = 34) and this was attributed to a group of islets (14 out of 59) with very low or negligible response (Fig. 5o, p). This is consistent with the islet heterogeneity in the nulls demonstrated by variable levels of NKX6.1, PDX1 and C-PEP immunostaining (ESM Fig. 4 e–h). Thus, reduced Ca^2+^ mobilisation contributes to, but is not solely responsible for, the observed phenotype.

**Fig. 6:**
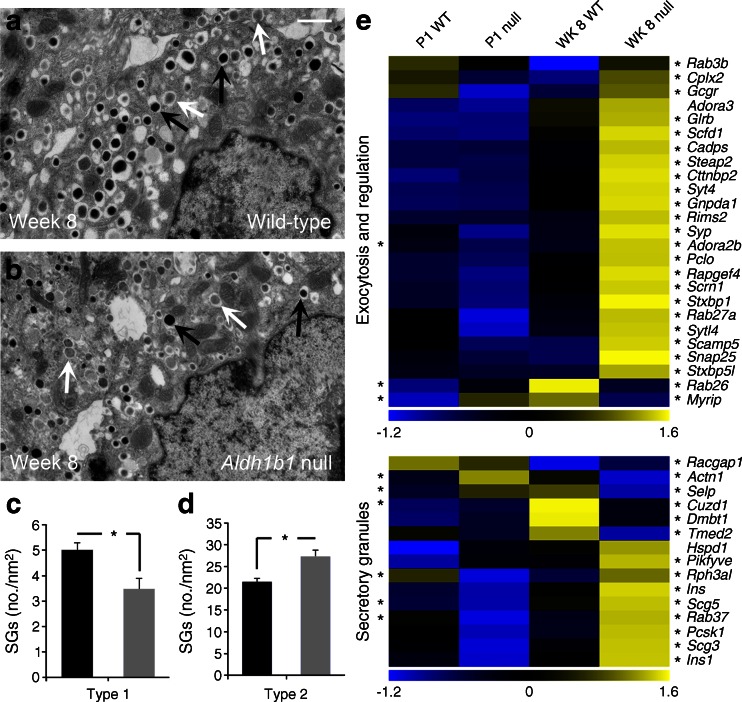
Immature secretory granules and misregulated exocytosis and SG genes in the *Aldh1b1* ^tm1lacZ^ null islets. (**a**–**d**) Transmission electron microscopy revealed that affected *Aldh1b1* ^tm1lacZ^ null beta cells have 30% (**c**) fewer type 1 SGs (mature granules shown by black arrows in **a**, **b**) and 22% (**d**) more type 2 SGs (immature granules shown by white arrows in **a**, **b**). Results are from three animals per genotype and islet cells were scored in double blind experiments for granule number and type (black bars, wild-type; grey bars, null). (**e**) *z* score heat maps showed misregulated expression of several genes involved in SG formation and exocytosis. Values are means ± SEM. *p < 0.01 for indicated comparison. Heat map, *p_adj_ ≤ 0.05: at the left side of the heat map * refers to comparisons at P1 whereas at the right side it refers to comparisons at WK 8. Scale bar, 1 μm. WK, week; WT, wild-type

Regulated insulin exocytosis is the final step of the stimulus-coupled insulin secretion. Underdeveloped or defective insulin SGs lead to compromised insulin secretion and glucose intolerance [42]. RNA Seq data showed an extensive deregulation of genes associated with cytoplasmic vesicle and SG biogenesis, already apparent at P1 and exacerbated at week 8. A large number of genes associated with SG docking, regulated exocytosis and GTPase regulation were misregulated at week 8 (Fig. 6e and ESM Fig. 5c, d, ESM Tables 1 and 2). Accordingly, we examined the numbers and morphology of insulin SGs in beta cells of *Aldh1b1*^tm1lacZ^ null and control mice at week 8 using transmission electron microscopy. Electron-dense dark SGs were scored as mature (type 1) and light-grey SGs as immature (type 2) [43] (Fig. 6a, b). Affected *Aldh1b1*^tm1lacZ^ null beta cells contained a slightly higher total number of SGs but, importantly, the ratio of immature to mature SGs was increased by more than twofold (Fig. 6c, d). Since the insulin-to-proinsulin ratio was not affected in the nulls this was most likely due to changes in their constitution and biogenesis.

Taken together, these data show that the functionality of the stimulus-coupling insulin secretion machinery of *Aldh1b1*^tm1lacZ^ null beta cells is impaired at multiple levels.

### Deterioration of glucose homeostasis with age

To understand the substantial hyperglycaemia that developed with age, we analysed mice at week 52. Glucose tolerance had further deteriorated and acute insulin secretion was weak and monophasic (ESM Fig. 6 a, b). Energy depletion and oxidative stress were again detected in *Aldh1b1*^tm1lacZ^ null islets but not in controls. Additionally, increased cell death was now detected in all null islets but not in controls (ESM Fig. 6 c–h). Also, proinsulin processing was now less efficient in the *Aldh1b1*^tm1lacZ^ nulls and pancreas glucagon levels were increased (ESM Fig. 3 i, j). As a result of chronic hyperglycaemia the nulls developed insulin resistance by week 52 (ESM Fig. 3f). Thus, a combination of persisting defects, detected earlier, as well as cell death, defective proinsulin processing and insulin resistance, cause the exacerbation of the phenotype in old age.

## Discussion

Beta cell dysfunction is a clinical hallmark of the progression to type 2 diabetes [1]. Studies in humans and rodents have associated nutrient depletion or environmental insults during fetal growth with increased susceptibility to adult onset of metabolic disease and beta cell dysfunction but little is known about the implicated molecular players [13–15]. The *Aldh1b1*^tm1lacZ^ null mice provide a model whereby early defects in fetal and neonatal stages manifest later in islet functional defects and deterioration of glucose homeostasis, capturing aspects of type 2 diabetes patients. Early defects in null young adult mice (weeks 6–8) are manifested both molecularly and functionally. Similar islet heterogeneity is encountered in all mice examined and therefore the phenotype among mice is relatively uniform. The presence of normal islets mitigates the phenotype.

Since *Aldh1b1* is expressed only in pancreatic progenitors and briefly in newly formed fetal beta cells, the origin of the documented islet defects is embryonic. The relatively mild phenotype could be due to the robust expression of other ALDH genes including mitochondrial ones in the developing pancreas (ESM Table 3). Islet heterogeneity may result because either compensating ALDH genes are not homogeneously expressed in the progenitors or shifting the differentiation window of endocrine cells at an earlier time deprives a subset of differentiating cells from essential signals.

Reduced expression of several transcription factors that play overlapping roles in establishing and maintaining beta cell functionality [5, 6, 8–12, 27, 44] was significant at P1 in the islets of *Aldh1b1*^tm1lacZ^ null mutants whereas misregulation of other beta cell functional components was manifested mostly later. Expression of these transcription factors was subsequently upregulated in the islets of *Aldh1b1*^tm1lacZ^ null adults suggesting an adaptive response. The increase in beta cell mass is also such a response but does not address the functional defects arising from the misregulation of essential transcription factors. Thus, misregulation of the beta cell transcription factor network is the primary result of *Aldh1b1* functional inactivation and underlies the multiplicity of defects in the islets of *Aldh1b1*^tm1lacZ^ null mice.

Availability of low ROS concentrations appears to be critical for self-renewal of some tissue stem cells [45, 46]. ALDH1B1 activity may participate in ROS level regulation in the pancreatic progenitors and, therefore, the regulation of the timing of lineage commitment. Premature commitment may limit the exposure of progenitors to necessary patterning signals. Alternatively, ALDH1B1 may modulate metabolic processes in the mitochondrion and, therefore, affect the levels of key metabolites necessary for the enzymes employed in epigenetic regulation of gene expression [47]. It has indeed been hypothesised that nutrient availability during fetal growth may lead to epigenetic changes predisposing to diabetes later in life [48]. Metabolomics of pancreatic progenitors and analysis of epigenetic changes in newly formed beta cells may clarify the underlying molecular mechanism.

In summary, the findings suggest that deregulation οf the transcription factors that control beta cell specification, maturation and maintenance is the main early effect of *Aldh1b1* inactivation and this occurs at the level of progenitor/committed endocrine cells during development. This has implications for the conversion of pluripotent stem cells into functional mature beta cells and the restoration of dysfunctional beta cells in type 2 diabetes. In addition, the findings indicate that genetic predisposition to type 2 diabetes may arise from mutations in developmental genes, which may not necessarily result in an early postnatal phenotype but manifest later in life.

## Electronic supplementary material

ESM Methods (PDF 146 kb)

ESM Fig. 1 (PDF 586 kb)

ESM Fig. 2 (PDF 621 kb)

ESM Fig. 3 (PDF 861 kb)

ESM Fig. 4 (PDF 1059 kb)

ESM Fig. 5 (PDF 668 kb)

ESM Fig. 6 (PDF 709 kb)

ESM Table 1 (PDF 81 kb)

ESM Table 2 (PDF 92 kb)

ESM Table 3 (PDF 39 kb)

## Abbreviations

ALDH: Aldehyde dehydrogenase
AMPK: AMP-activated protein kinase
AMY: Amylase complex
C-PEP: C-peptide
DBA: Dolichos biflorus agglutinin
dpc: Days post coitum
GCG^+^: Glucagon-positive
GLP1R: Glucagon-like peptide-1 receptor
GSIS: Glucose-stimulated insulin secretion
IPGTT: Intraperitoneal glucose tolerance test
IPITT: Intraperitoneal insulin tolerance test
IPPTT: Intraperitoneal pyruvate tolerance test
NGN3: Neurogenin 3
NKX6.1: NK6 homeobox 1
P1: Postnatal day 1
PDX1: Pancreatic and duodenal homeobox 1
PH3: Phosphohistone-H3
ROS: Reactive oxygen species
SG: Secretory granule
SOM: Somatostatin
