# Supplementary material for: Aldehyde dehydrogenase activity is necessary for beta cell development and functionality in mice
Source: Diabetologia. 2015 Oct 31;59(1):139–50. doi: 10.1007/s00125-015-3784-4 (PMC4670456; doi:10.1007/s00125-015-3784-4)
Supplement: Supplementary file 1 — (PDF 146 kb) [file 125_2015_3784_MOESM1_ESM.pdf]

ELECTRONIC SUPPLEMENTARY MATERIAL (ESM)

ESM Methods

***Aldh1b1^{tm1lacz}* Knock In Mouse Strain.**

Targeted mouse *Aldh1b1$^{tm1(KOMP)Vlcg}$* ES cells (Figure S1) where the *lacz* cDNA was inserted in the *Aldh1b1* locus under the control of the cognate promoter were expanded and injected in C57BL/6J-Tyr blastocysts using standard procedures. The resulting chimeric mice were bred with C57/Bl6J mice thus maintaining the line in an isogenic background. Following germline transmission the *PGK-Neo* selection cassette was removed after intercrossing with the *Tg$^{Hs\text{-}Cre1}$* transgenic C57/Bl6J line (1) thus establishing the *Aldh1b1^{tm1lacz}* mouse line (Figure S1). The line was expanded through interbreeding with C57/Bl6J wild type mice excluding individuals carrying the *Tg$^{Hs\text{-}Cre1}$* allele. Genotyping by qPCR for at least two generations was used to ensure removal of the selection cassette and the absence of the *Hs-Cre1* allele. We then established that in the developing pancreas of heterozygous embryos, *Aldh1b1* expression was accompanied by β-galactosidase expression and confirmed the absence of ALDH1B1 expression in the developing homozygous pancreata (Figure S1D-G). Homozygotes were born in the expected mendelian ratio and were viable and fertile. Genotyping procedures were as described for *Aldh1b1* alleles (http://www.velocigene.com/komp/detail/11807), and *Cre* (2). Animal maintenance and experimentation were in accordance with international guidelines and subjected to ethical approval from the competent veterinary committees of Athens and TU Dresden.

Insulin Tolerance (IPITT) and Pyruvate Tolerance (IPPTT) tests

For insulin tolerance tests (IPITT), mice were fasted for 4 hours and injected intraperitoneally with insulin (Humulin; Eli Lilly) at 0.4 U/Kg body weight. Tail vein blood samples were collected at various time-points after injection, and glucose

concentration was determined using the Contour XT blood glucose monitoring system (Bayer). For pyruvate tolerance tests (IPPTT), mice were fasted for 16 hours overnight and injected intraperitoneally with Sodium Pyruvate (Sigma) at 1.5g/Kg body weight. Tail vein blood samples were collected at various time points after injection, and glucose concentration was determined using the Contour XT system.

Immunostainings and Morphometric Analysis

Morphometric analysis

All morphometric analyses of embryonic, newborn and adult pancreata were performed using immunofluorescent images taken at saturation and the ImageJ software as described previously (2). Embryonic pancreata were analyzed using 12-μm-thick cryosections at least four sections apart and covering the entire tissue. For β-cell mass quantitation, the signal area of C-PEP-positive immunofluorescence was calculated and divided by the corresponding total signal area for DAPI, thus normalizing for pancreatic mass. To visualize all β-cells in the null samples due to reduced C-PEP staining area quantitation was done following image acquisition at increased exposure. For epithelial mitotic quantitation, PH3-positive nuclei were counted and divided over the epithelial DAPI area as marked by E-CADHERIN co-staining. For endocrine progenitor quantitation, NGN3-positive nuclei were counted and divided over total DAPI area. Adult pancreata were analysed using 12-μm-thick cryosections at least 120μm apart, so that any given islet was scored only once. For islet mitosis, PH3-positive nuclei were counted and divided over C-peptide area. For α- and δ-cell mass quantitation, the signal area of GLUCAGON- and SOMATOSTATIN-positive immunofluorescence respectively was calculated and divided by the corresponding islet DAPI area. Finally, for islet size distribution analysis, islet sizes were calculated using C-PEP immunofluorescence as described (2), and distributions were expressed as the probability density function. For each quantitation at least three pancreata of each genotype were analyzed.

Antibodies used for Immunofluorescence

Primary antibodies used were: rabbit anti-NGN3 (1:100; Acris), rat anti-E-CADHERIN (1:400; Zymed), rabbit anti-SOX9 (1:500; Chemicon), rabbit anti-C-PEPTIDE (1:200; Linco), DBA-Rhodamine lectin (1:100; Vector), rabbit anti-AMYLASE (1:300; Sigma), mouse anti-GLUCAGON (1:500; Sigma), rabbit anti-PDX1 (1:5000; Gift from C. Wright), mouse anti-NKX6-1 (1:1000; DSHB), mouse anti-INSULIN (1:1000; Sigma), rabbit anti-PH3 (1:500; Cell Signaling), rabbit anti-SOMATOSTATIN (1:200; Zymed), rabbit anti-CASPASE-3 (1:40; Millipore), rabbit anti-PAMPKα (1:100; Cell Signailing), mouse anti-4-HNE (1:50; Abcam), rabbit anti-ALDH1B1 (1:200; Proteintech and raised in MPI-CBG with a described epitope (3), rabbit anti mouse anti-B-GALACTOSIDASE (1:250; Promega), rabbit anti-GLP1R (1:1000; Gift from S. Heller) and rabbit anti-GLUT2 (1:500; Millipore). Immunostainings with anti-4-HNE required antigen retrieval by microwaving slides at 600W for 4 minutes in citrate buffer (0.01M citric acid, pH6.0). Immunostainings with anti-PAMPKα required signal amplification with the TSA system (Perkin Elmer), performed according to the manufacturer's instructions. Secondary antibodies were anti-mouse, anti-rabbit and anti-rat Alexa-488-, Alexa-568- and Alexa-633-conjugated goat antibodies (1:500; Molecular Probes). Primary antibodies were validated against tissue not expressing the antigen in question and secondary antibodies were validated in the absence of primary antibodies.

Islet isolation and functional analyses

Islet isolation and Insulin Secretion Assays

Islets were hand-picked in RPMI-1640 medium (Sigma) supplemented with 10% FBS (Sigma) and 20mM L-glutamine (Gibco). For GSIS assays, islets of similar size were transferred in a round-bottom 96-well plate at 5 islets/well (at least 5 groups per animal), in 200μl Extracellular Solution (ECS: 125mM NaCl, 2.5mM KCl, 26mM $NaHCO_3$, 1.25mM NaH_2PO_4, 1mM $MgCl_2$, 2mM $CaCl_2$ and 10mM Hepes pH 7.4) (4)

supplemented with 3mM glucose and incubated for 1 hour at 37°C and 5% CO_2. Medium was replaced with fresh ECS containing 3mM D-glucose (baseline medium) and islets were incubated for 1 hour at 37°C and 5% CO_2. Baseline medium was then replaced with stimulatory medium (ECS containing 20mM D-glucose) for another hour at 37°C and 5% CO_2. Insulin secreted in the baseline and stimulatory media was measured using a mouse insulin ELISA (Mercodia) or an ultrasensitive mouse insulin ELISA (Mercodia). For drug-stimulated insulin secretion assays at least 4 groups of islets were measured. The stimulatory medium was ESC containing 3mM D-glucose and either 100μM Tolbutamide (Sigma) or 2μM BayK8644 (Sigma).

Measurements of islet intracellular calcium concentration $[Ca^{2+}]_i$ changes

Changes in $[Ca^{2+}]_i$ of isolated islets from *Aldh1b1*^{tm1lacz} null and wt mice were performed by epifluorescence imaging. Islets were embedded in fibrin gel (2 mg/ml fibrinogen, 10 U/ml thrombin in HBSS; Sigma-Aldrich) for fixation. After gel polymerization, islets were loaded with the Ca^{2+} indicator Oregon Green 488 BAPTA-1 AM (Life Technologies) at 1.5 μM in ECS containing 3 mM glucose and 0.01% Pluronic F-127 (life technologies) for 2 hours at 37°C. For measurement of stimulus-induced $[Ca^{2+}]_i$ changes, fixed islets were mounted on an upright microscope equipped with an 20x 1.0 water immersion objective. Islets were perfused with ECS containing 3 mM glucose for baseline conditions and 20 mM for stimulation. Imaging was performed with an epifluorescence microscope (Zeiss Axio Examiner A1) with a Cairn Monochromator OptoScan. Islets were excited at 488nm and images were taken with a frequency of 5 seconds and an exposure time of 0.05 seconds using a Clara CCD iXon3 camera (Andor, UK). Images were analyzed by Andor iQ 2.7 software (Andor, UK, 2002-2005).

ROS detection using fluorescence

Islets were transferred in 8 well 15μ-Plates (Ibidi) embedded in fibrin gel at 4 islets/well (at least 4 groups per animal, 3 animals per genotype) in 300ul ECS

supplemented with 10mM glucose and placed at 37 ^{o}C, 5% CO_2 for 30 min. Then, CM-H2DCFDA (Life Technologies) was added in the wells to a final concentration of 20 uM and islets were placed back in the incubator for another 30 min. Finally, islets were quickly rinsed with dye-free ECS twice, and imaged in the same medium using a Leica SP5 confocal microscope.

ATP Measurements

ATP measurements on isolated islets were performed as described previously using the ENLITEN ATP Assay kit (Promega). Protein levels were quantitated using the QuantiPro BCA Assay kit (Sigma) and results were expressed as moles of ATP per µg protein.

Transmission Electron Microscopy

Islets were isolated from WK8 male mice, fixed and processed for image acquisition as described previously (5). Islet cells were scored in double blind experiments for granule number and type as well as cell area using the Fiji software. Results were expressed as the ratio of mature or immature granules over β-cell area.

Real-Time PCR, RNA Seq and bioinformatics analyses

RNA sequencing and bioinformatic analyses

Islets were isolated from P1 and male WK 8 mice. For P1, islets from three or more pups of the same genotype (*Aldh1b1*^{tm1lacz} null or wt) were pooled to generate one sample. Three independent samples from each stage and genotype were used as biological replicates. Total RNA with an integrity number of ≥ 8 was used. mRNA was isolated from 1ug total RNA by poly-dT enrichment using the NEBNext Poly(A) mRNA Magnetic Isolation Module according to the manufacturer's instructions. Final elution was done in 15ul 2x first strand cDNA synthesis buffer (NEBnext, NEB). After chemical fragmentation by incubating for 15 min at 94°C the sample was directly

subjected to the workflow for strand specific RNA-Seq library preparation (Ultra Directional RNA Library Prep, NEB). For ligation custom adaptors were used (Adaptor-Oligo 1: 5'-ACA-CTC-TTT-CCC-TAC-ACG-ACG-CTC-TTC-CGA-TCT-3', Adaptor-Oligo 2: 5'-P-GAT-CGG-AAG-AGC-ACA-CGT-CTG-AAC-TCC-AGT-CAC-3'). After ligaton adapters were depleted by an XP bead purification (Beckman Coulter) adding bead in a ratio of 1:1. Indexing was done during the following PCR enrichment (15 cycles) using custom amplification primers carring the index sequence indicated with 'NNNNNN'. (Primer1: Oligo_Seq AATGATACGGCGACCACCGAGATCTACACTCTTTCCCTACACGACGCTCTTCCG ATCT, primer2: GTGACTGGAGTTCAGACGTGTGCTCTTCCGATCT, primer3: CAAGCAGAAGACGGCATACGAGAT NNNNNN GTGACTGGAGTT. After two more XP beads purifications (1:1) libraries were quantified using Qubit dsDNA HS Assay Kit (Invitrogen). For Illumina flowcell production, samples were equimolarly pooled and distributed on all lanes used for 75bp single read sequencing on Illumina HiSeq 2500. After sequencing, FastQC (http://www.bioinformatics.babraham.ac.uk/) was used to perform a basic quality control on the resulting reads. As an additional control, library diversity was assessed by redundancy investigation in the mapped reads. Alignment of the short reads to the mm9 transcriptome was performed with pBWA (7) and a table of readcounts per gene was created based on the overlap of the uniquely mapped reads with the Ensembl Genes annotation v. 67 (May 2012) for mm9, using BEDtools (v. 2.11) (8). Normalization of the raw read counts based on the library size and testing for differential expression between the different cell types/treatments was performed with the DESeq R package (v.1.10.1) (9). Sample to sample Euclidean distance as well as Pearson's correlation coefficient (r) were computed based on the normalized gene expression level in order to explore correlation between biological replicates and different libraries. For testing for differential expression, the count data were fitted to the negative binomial distribution and the p-values for the statistical significance of the fold change were adjusted for

multiple testing with the Benjamini-Hochberg correction for controlling the false discovery rate (10). Accepting a maximum of 10% false discoveries (padj ≤ 0.1) for each time point genes with normalized counts > 50 in either nulls or controls and fold change < 0.6 or > 1.6 were considered regulated. GO analyses were done using the DAVID suite (http://david.abcc.ncifcrf.gov/). Heat maps for regulated genes with normalised counts ≥ 100 in at least one time point and condition were generated by calculating the z score for each gene in each sample across both time points (z score = (normalized gene counts - mean of normalized counts) / standard deviation). Gene lists with the average gene z scores for each time point and genotype, were introduced in the MeV software and average linkage hierarchical clustering was used to generate the heat maps.

Real Time PCR

Liver samples were dissected and islets were isolated from 20-week old mice fasted for 4 hours. Total RNA isolation and first-strand cDNA preparation were according to standard procedures (11). Real Time PCR primers were designed using the Primer 3 software (SimGene), specificity was ensured by *in silico* PCR, reactions were performed with SYBR-Greener (Invitrogen) using an ABI PRISM 7000 machine and primary results were analysed using its software. Reactions were carried out from four independent samples. Absolute expression values were calculated using the ΔCt method as described previously (11). Primers used were further evaluated by inspection of the dissociation curve and primers were G-6-Pase (F) GAGGTACCAAGGGAGGAAGG, G-6-Pase (R) TGGAACCAGATGGGAAAGAG, Actin (F) TGGCTCCTAGCACCATGA and Actin (R) CCACCGATCCACACAGAG.

Hormone measurements and liver glycogen content assay

INSULIN, PROINSULIN and GLUCAGON measurements

To determine pancreatic INSULIN, PROINSULIN and GLUCAGON content, the pancreas was homogenized in 5ml Acid-Ethanol (0.18M HCl in 70% ethanol) using a Polytron homogenizer. After an overnight incubation at -20°C and a brief centrifugation at 2000rpm for 15 minutes, the supernatant was neutralized with an equal volume of 1M Tris pH7.5. The pellet was taken through a second cycle of homogenization, freezing and centrifugation, and the neutralized supernatants were finally combined. Insulin, proinsulin and glucagon levels were quantitated using ELISAs specific for the corresponding mouse hormones (all from Mercodia) and using appropriate dilutions according to the manufacturer's protocol. Results were normalized to total protein, measured using the QuantiPro BCA Assay kit (Sigma). For hormone measurements in serum, whole blood was collected from the submandibular vein, allowed to clot by standing for 30 minutes at room temperature and centrifuged at 13000rpm for 12 minutes to pellet cells. Hormone levels were assayed in the supernatant using the appropriate ELISA kit (Mercodia).

Liver glycogen content assay

Liver samples were dissected from 20-week old mice and flash-frozen in liquid nitrogen. Samples weighing approximately 20mg were transferred in 0.5ml of pre-heated HCl (2M) and left in boiling water for 1 hour. To achieve complete hydrolysis, liver samples were shaken vigorously every 10 minutes during boiling. After cooling down to room temperature, the hydrolysis product was neutralized with 0.5ml of NaOH (2M) and samples were centrifuged at maximum speed for 10 minutes. Glucose levels in the supernatant were determined using the Glucose (HK) Assay kit (Sigma) according to the manufacturer's instructions. Results were expressed as μg glucose per mg liver.

Western blotting and X-gal stainings

Western blotting

Pancreata dissected from 14.5-dpc mouse embryos and islets isolated from 20-week old wild type mice were used. Protein extraction, polyacrylamide gel electrophoresis and blotting procedures were according to standard protocols. Primary antibodies used were: rabbit anti-ALDH1B1 (1:5000; raised in MPI-CBG with a described epitope (3)) and mouse anti B-ACTIN (1:5000; Santa Cruz). Secondary antibodies were anti-rabbit and anti-mouse horseradish peroxidase (HRP)-conjugated goat antibodies (1:5000; Dako).

X-gal stainings

Whole 9.5 dpc embryos were fixed in 2% PFA for 15 minutes at room temperature and stained with 5-bromo-4-chloro-3-indolyl-β-D-galactopyranoside (X-Gal; Roche) as described previously (6). Images were acquired using a Nikon stereoscope (WD70), fitted with a color camera (Leica DFC320).

ESM references

1. Dietrich P, Dragatsis I, Xuan S, Zeitlin S, Efstratiadis A: Conditional mutagenesis in mice with heat shock promoter-driven cre transgenes. Mamm Genome 2000;11:196-205
2. Serafimidis I, Heximer S, Beis D, Gavalas A: GPCR signaling and S1P play a phylogenetically conserved role in endocrine pancreas morphogenesis. Mol Cell Biol 2011;31:5702-5711
3. Stagos D, Chen Y, Brocker C, Donald E, Jackson BC, Orlicky DJ, Thompson DC, Vasiliou V: Aldehyde dehydrogenase 1B1: molecular cloning and characterization of a novel mitochondrial acetaldehyde-metabolizing enzyme. Drug Metab Dispos 2010;38:1679-1687
4. Speier S, Yang SB, Sroka K, Rose T, Rupnik M: KATP-channels in beta-cells in tissue slices are directly modulated by millimolar ATP. Mol Cell Endocrinol 2005;230:51-58
5. Knoch KP, Bergert H, Borgonovo B, Saeger HD, Altkruger A, Verkade P, Solimena M: Polypyrimidine tract-binding protein promotes insulin secretory granule biogenesis. Nat Cell Biol 2004;6:207-214
6. Gu C, Stein GH, Pan N, Goebbels S, Hornberg H, Nave KA, Herrera P, White P, Kaestner KH, Sussel L, Lee JE: Pancreatic beta cells require NeuroD to achieve and maintain functional maturity. Cell Metab 2010;11:298-310
7. Peters D, Luo X, Qiu K, Liang P: Speeding Up Large-Scale Next Generation Sequencing Data Analysis with pBWA. J Biocomput 2012;1:1
8. Quinlan AR, Hall IM: BEDTools: a flexible suite of utilities for comparing genomic features. Bioinformatics 2010;26:841-842
9. Anders S, Huber W: Differential expression analysis for sequence count data. Genome Biol 2010;11:R106
10. Benjamini Y, Hochberg Y: Controlling the false discovery rate: a practical and powerful approach to multiple testing. J Roy Stat Soc B 1995;57:289-300
11. Ioannou M, Serafimidis I, Arnes L, Sussel L, Singh S, Vasiliou V, Gavalas A: ALDH1B1 is a potential stem/progenitor marker for multiple pancreas progenitor pools. Dev Biol 2013;374:153-163
