# Supplementary material for: Aldehyde dehydrogenase activity is necessary for beta cell development and functionality in mice
Source: Diabetologia. 2015 Oct 31;59(1):139–50. doi: 10.1007/s00125-015-3784-4 (PMC4670456; doi:10.1007/s00125-015-3784-4)
Supplement: Supplementary file 2 — (PDF 586 kb) [file 125_2015_3784_MOESM2_ESM.pdf]

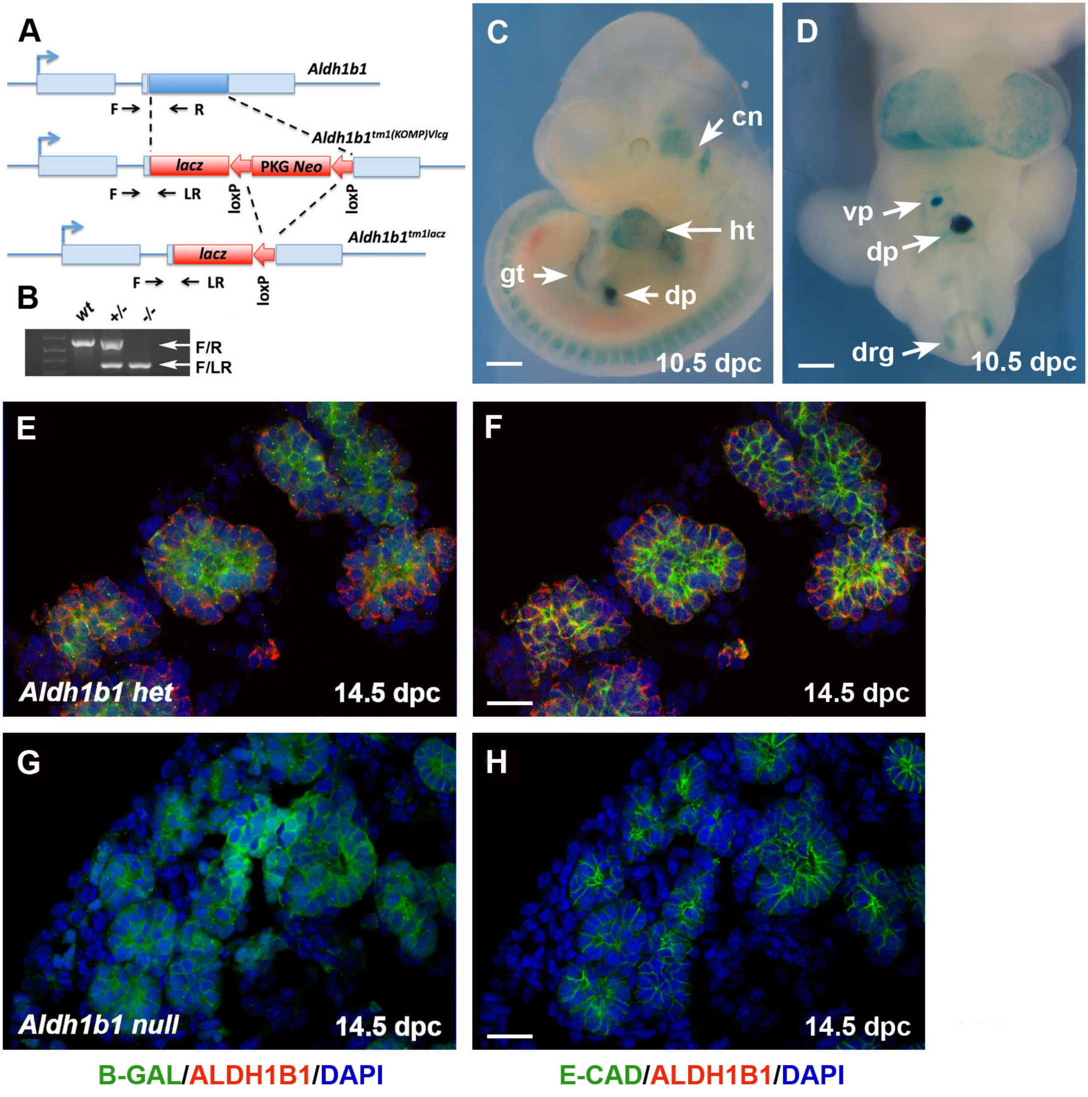

Anastasiou et al, ESM Fig 1

ESM Fig. 1. Generation of the *Aldh1b1*^{tm1lacz} knock in mouse strain

(A, B) Schematic representation of the targeting of *Aldh1b1* genetic locus with a *lacZ-PKG-Neo* transgene to generate the *Aldh1b1*$^{tm1(KOMP)Vlcg}$ ES cells and mouse line. The *PKG-Neo* cassette was removed after intercrossing with the *Tg*$^{Hs\text{-}Cre1}$ transgenic line, thus establishing the *Aldh1b1*^{tm1lacz} mouse line.

(C, D) X-gal staining for β-galactosidase on *Aldh1b1*^{tm1lacz} heterozygote E10.5 embryos confirms that *Aldh1b1* is expressed in the developing dorsal (dp) and ventral (vp) pancreatic buds. There is also expression in the heart (ht), cranial nerves (cn), dorsal root ganglia (drg) and the gut (gt).

(E-H) Double immunofluorescence for B-GAL and ALDH1B1 (E, G) or E-Cadherin and Aldh1b1 (F, H) in pancreata from E14.5 *Aldh1b1*^{tm1lacz} heterozygote (E, F) or null (G, H) embryos confirms the absence of ALDH1B1 in the developing null pancreata. Scale bars, (C) 500µm, (D) 250µm, (E-H) 25µm.
