# Supplementary material for: Aldehyde dehydrogenase activity is necessary for beta cell development and functionality in mice
Source: Diabetologia. 2015 Oct 31;59(1):139–50. doi: 10.1007/s00125-015-3784-4 (PMC4670456; doi:10.1007/s00125-015-3784-4)
Supplement: Supplementary file 3 — (PDF 621 kb) [file 125_2015_3784_MOESM3_ESM.pdf]

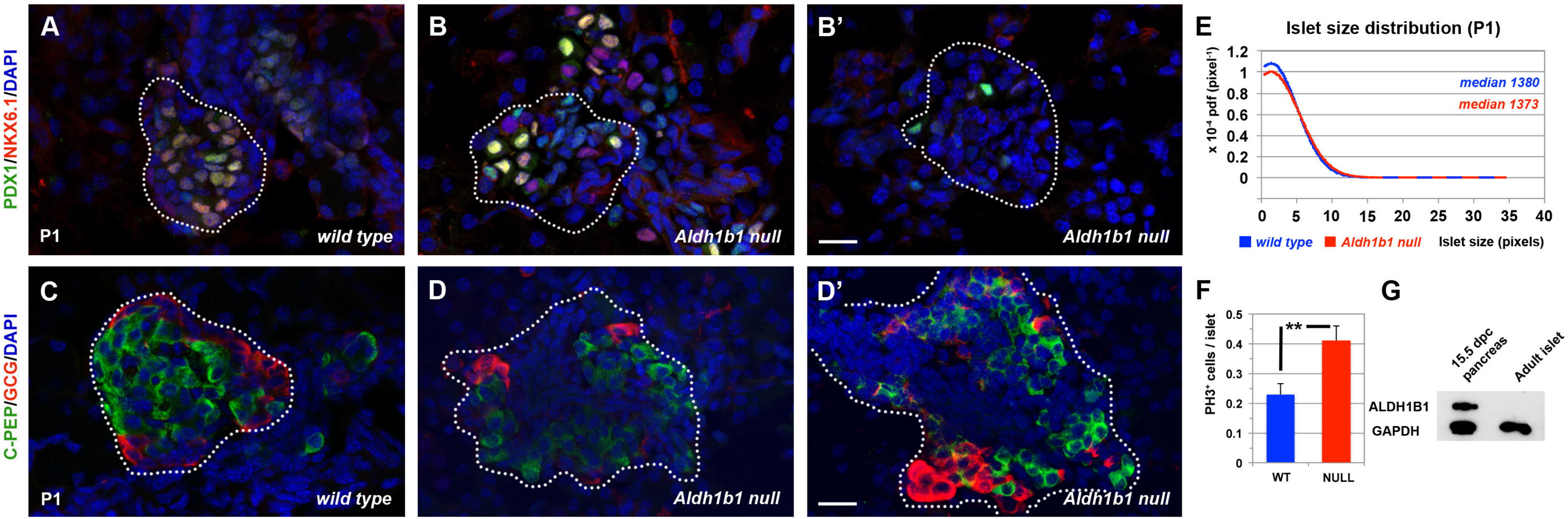

Anastasiou et al, ESM Figure 2

ESM Fig. 2. Islet patterning is defective in newborn *Aldh1b1^{tm1lacz}* null mice; ALDH1B1 protein is not detectable in the adult mouse islet.

(A, B, B') Double immunofluorescence for PDX1 and NKX6.1 revealed a striking heterogeneity in the *Aldh1b1^{tm1lacz}* P1 null islets (n=3).

(C, D, D') Double immunofluorescence for C-PEP and GCG showed variable levels of expression of these markers in *Aldh1b1^{tm1lacz}* null P1 islets (n=3).

(E) No change was observed in the median islet size between *Aldh1b1^{tm1lacz}* null and control P1 mice, as evident also by the probability density functions (pdf) for islet size. Sizes are expressed in pixels (100μm = 135 pixels)(n=3).

(F) Quantitation of PH3 immunofluorescence indicated that mitotic activity was 2-fold higher in *Aldh1b1^{tm1lacz}* null P1 islets compared to wild-type controls (n=3).

(G) At the protein level, ALDH1B1 is strongly expressed in the embryonic pancreas, however it is completely absent from the adult islet, as shown by western blotting on wild type E15.5 embryonic pancreata and isolated adult pancreatic islets.

Values in (F) are mean±SEM. *$p<0.05$, Scale bars, 20μm.
