# Supplementary material for: Aldehyde dehydrogenase activity is necessary for beta cell development and functionality in mice
Source: Diabetologia. 2015 Oct 31;59(1):139–50. doi: 10.1007/s00125-015-3784-4 (PMC4670456; doi:10.1007/s00125-015-3784-4)
Supplement: Supplementary file 4 — (PDF 861 kb) [file 125_2015_3784_MOESM4_ESM.pdf]

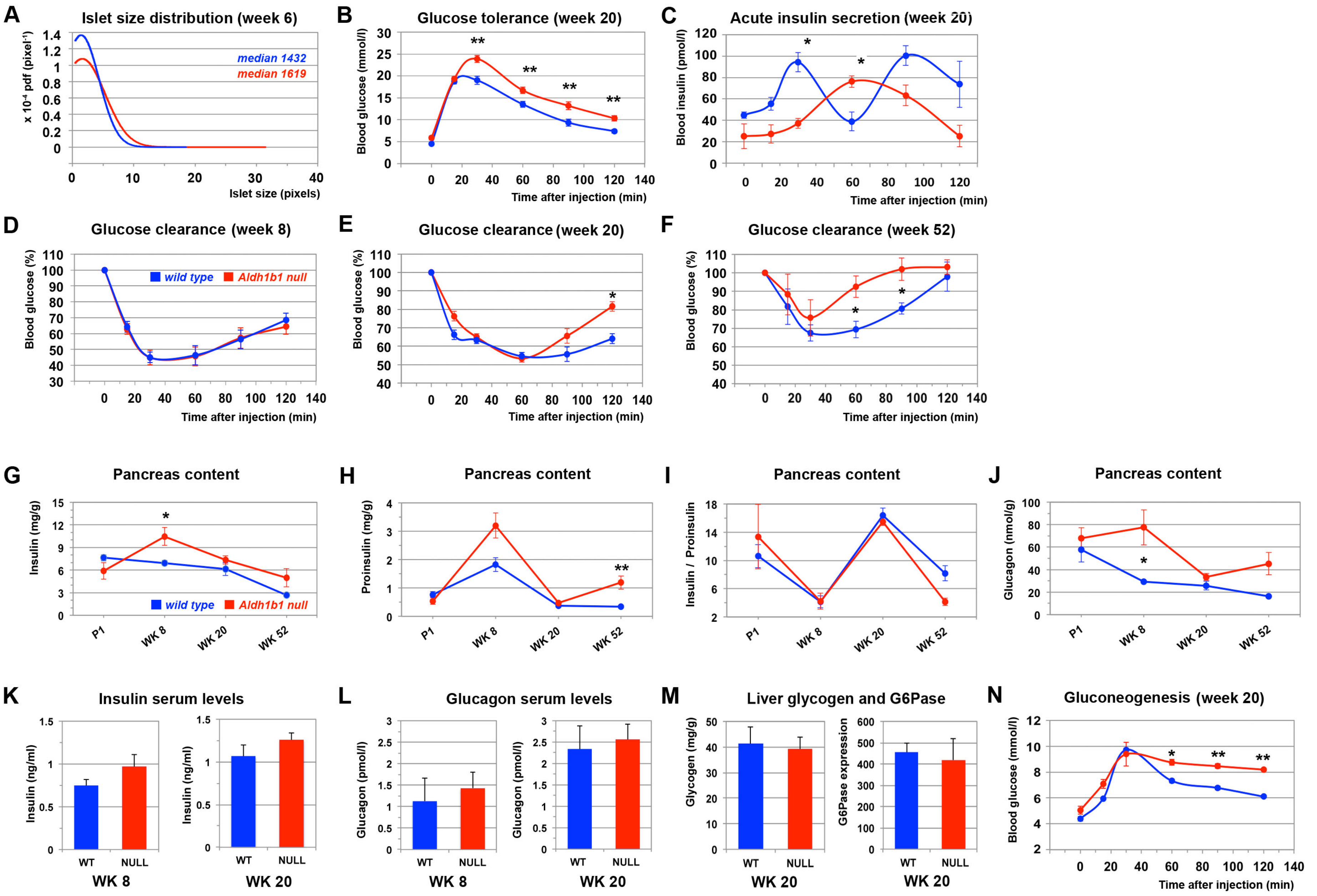

Anastasiou et al, ESM Figure 3

ESM Fig. 3. Collective metabolic data on P1, WK 8, 20 and 52 *Aldh1b1*^{tm1lacz} null and control mice

(A) Morphometric analysis and plotting of the islet size probability density function (pdf) illustrates the median islet size in wt and *Aldh1b1*^{tm1lacz} null mice at WK 6. Sizes are expressed in pixels (100μm = 135 pixels). Differences in the distribution are not significant.

(B) *Aldh1b1*^{tm1lacz} null mice show a significant delay in blood glucose clearance during IPGTT (n=8, 7) at WK 20.

(C) Acute insulin secretion during IPGTT is impaired in *Aldh1b1*^{tm1lacz} null mice at WK 20 (n=6).

(D-F) Normal rates of glucose clearance in WK 8 and 20 *Aldh1b1*^{tm1lacz} null mice during an IPIT test, however 52 WK null mice showed clear signs of insulin resistance (n=3, 7).

(G-J) Total pancreatic insulin (G), proinsulin (H), insulin / proinsulin ratios (I) and glucagon (J) levels remained relatively constant over time with the exception of hormone levels at WK 8 and the insulin/proinsulin ratio at WK 52 (n=3, 8).

(K, L) Serum insulin and glucagon levels following a 4 hr fasting period were similar between *Aldh1b1*^{tm1lacz} null and control mice both at 8 and 20 weeks of age (n=5, 9).

(M) Hepatic stores of glycogen as well as expression of liver G6Pase remain very similar between WK 20 *Aldh1b1*^{tm1lacz} null and control mice (n=3-5).

(N) Gluconeogenesis rates in WK 20 old *Aldh1b1*^{tm1lacz} nulls are not affected as evident by an IPPT test (n=3, 4).

Values are mean±SEM. *p<0.05, **p<0.01.
