# Supplementary material for: Aldehyde dehydrogenase activity is necessary for beta cell development and functionality in mice
Source: Diabetologia. 2015 Oct 31;59(1):139–50. doi: 10.1007/s00125-015-3784-4 (PMC4670456; doi:10.1007/s00125-015-3784-4)
Supplement: Supplementary file 5 — (PDF 1059 kb) [file 125_2015_3784_MOESM5_ESM.pdf]

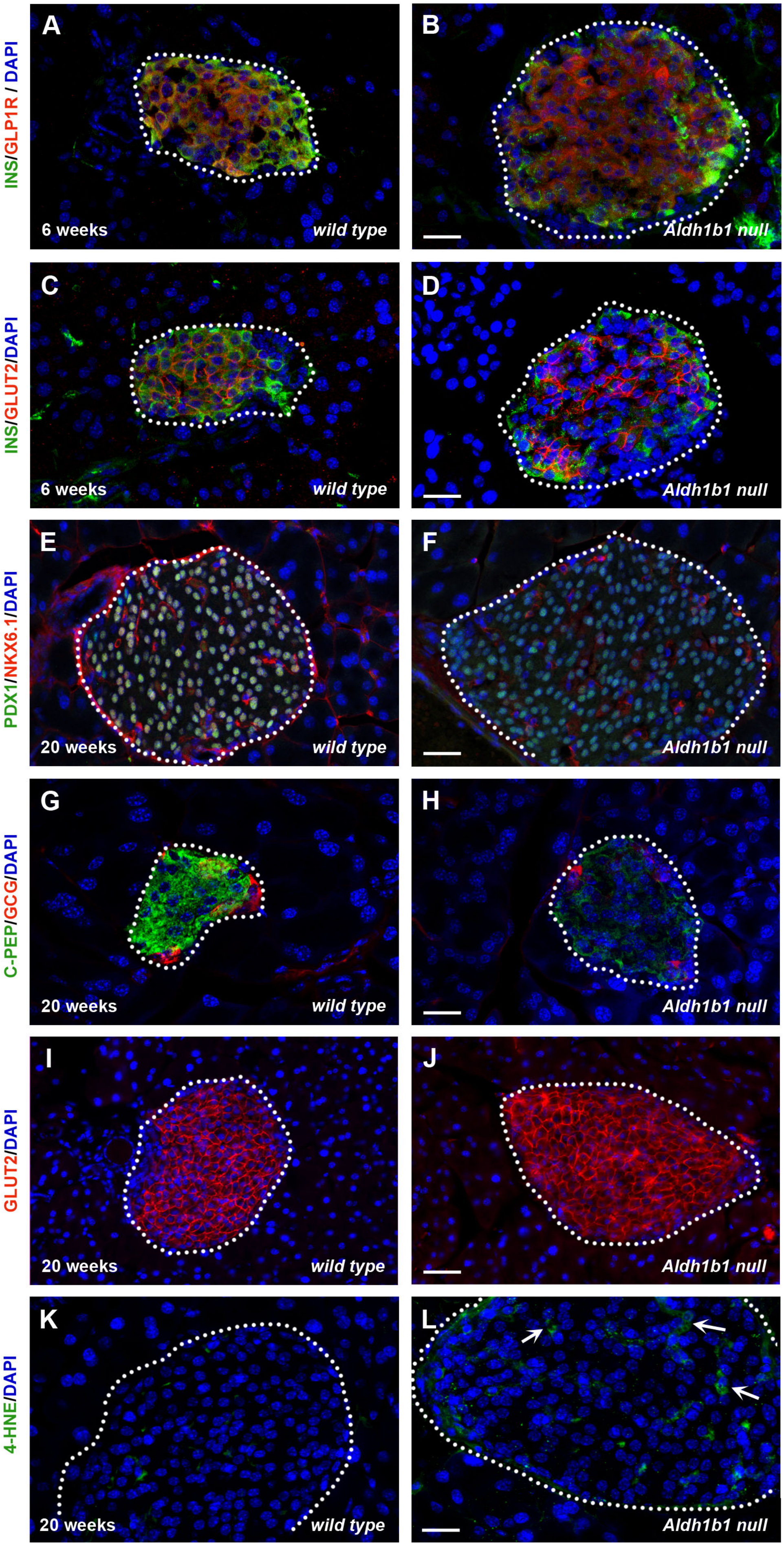

Anastasiou et al, ESM Figure 4

ESM Fig. 4. Expression of mature islet markers in WK6 and WK 20 *Aldh1b1*^{tm1lacz} null and control mouse pancreata

(A-D) Double immunofluorescence for INS and either GLP1R (A, B) or GLUT2 (C, D) revealed no differences between *Aldh1b1*^{tm1lacz} null and control WK 6 islets (n=3).

(E-H) Consistent with the early adult phenotype, double immunofluorescence for PDX1 and NKX6.1 (E, F) or C-PEP and GCG (G, H) revealed significant decrease in NKX6.1 and C-PEP expression in WK 20 *Aldh1b1*^{tm1lacz} null islets compared to controls (n=3).

(I, J) No difference was observed in expression of the glucose transporter 2 (GLUT2) between *Aldh1b1*^{tm1lacz} null and control WK 20 islets (n=3).

(K, L) *Aldh1b1*^{tm1lacz} null islets show increased oxidation of unsaturated fatty acid (arrows in L) (n=3).

Scale bars, (A-F) 25µm, (G, H) 20µm, (I, J) 50µm, (K, L) 20 µm
