# Supplementary material for: Aldehyde dehydrogenase activity is necessary for beta cell development and functionality in mice
Source: Diabetologia. 2015 Oct 31;59(1):139–50. doi: 10.1007/s00125-015-3784-4 (PMC4670456; doi:10.1007/s00125-015-3784-4)
Supplement: Supplementary file 6 — (PDF 668 kb) [file 125_2015_3784_MOESM6_ESM.pdf]

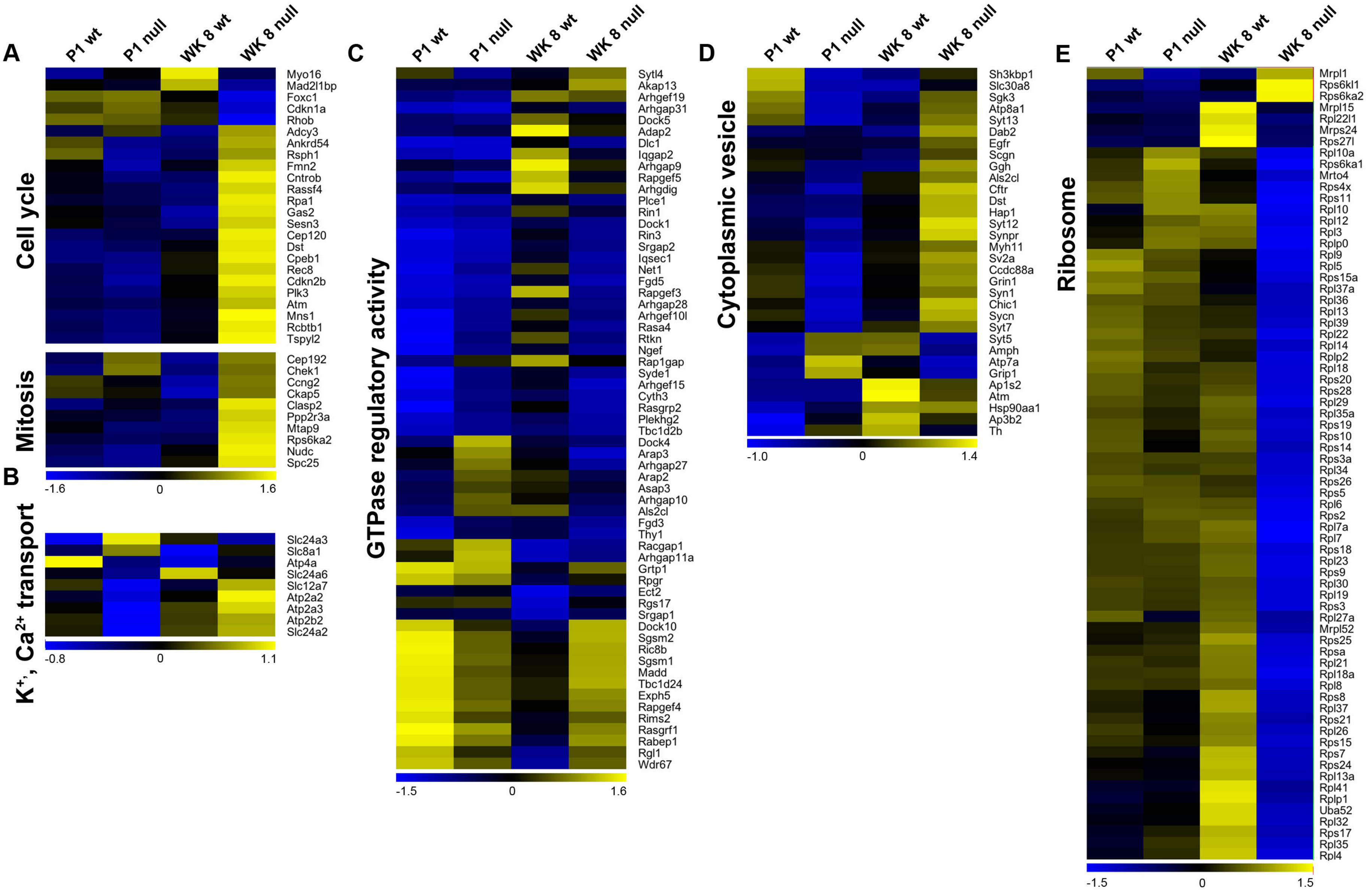

Anastasiou et al, ESM Figure 5

ESM Fig. 5. Heat maps of regulated genes involved in key processes of β-cell function and ontogeny

(A-E) Z-score heatmaps showed misregulated expression of several genes involved in cell cycle control and mitosis (A), K^+ and Ca^{2+} transport (B), GTPase regulatory activity (C) cytoplasmic vesicle formation (D) and ribosome biogenesis (E).
