# Supplementary material for: Aldehyde dehydrogenase activity is necessary for beta cell development and functionality in mice
Source: Diabetologia. 2015 Oct 31;59(1):139–50. doi: 10.1007/s00125-015-3784-4 (PMC4670456; doi:10.1007/s00125-015-3784-4)
Supplement: Supplementary file 7 — (PDF 709 kb) [file 125_2015_3784_MOESM7_ESM.pdf]

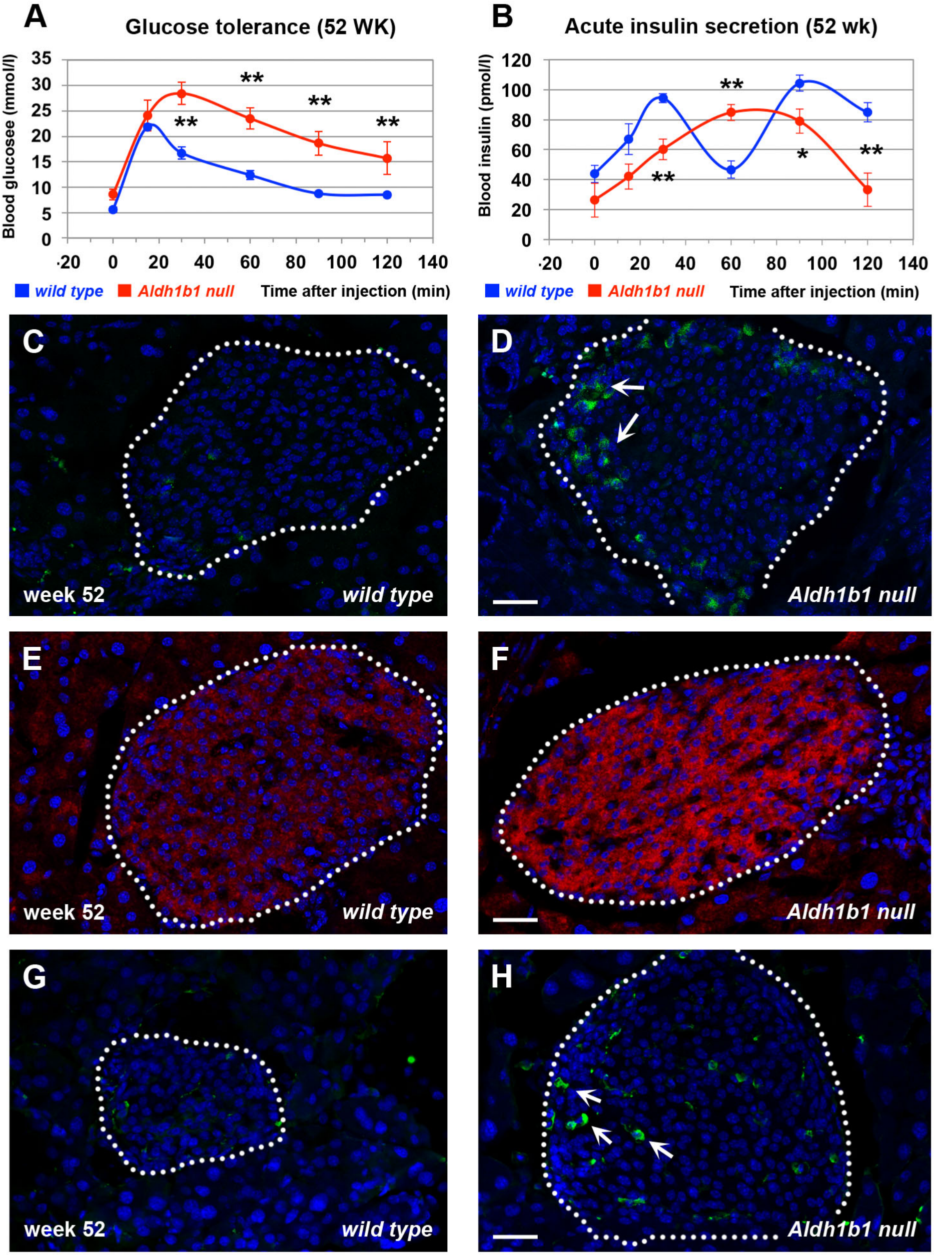

Anastasiou et al, ESM Figure 6

ESM Fig. 6. Phenotypic analysis of aged (52-week old) *Aldh1b1^{tm1lacz}* null mice

(A) IPGTT on WK 52 *Aldh1b1^{tm1lacz}* null and control mice show a significant delay in blood glucose clearance in the null (n=3, 4).

(B) Acute insulin secretion during IPGTT is impaired in WK 52 *Aldh1b1^{tm1lacz}* null mice (n=3, 4).

(C, D) TUNEL staining indicates increased levels of apoptosis in WK 52 *Aldh1b1^{tm1lacz}* null islets (n=3).

(E, H) Immunofluorescence for P-AMPKA (E, F) and 4-HNE (G, H) revealed that 52 WK *Aldh1b1^{tm1lacz}* null islets are energy depleted and show signs of oxidative stress.

Values are mean ±SEM. *$p<0.05$. Scale bars, 25μm.
