# Supplementary material for: Aldehyde dehydrogenase activity is necessary for beta cell development and functionality in mice
Source: Diabetologia. 2015 Oct 31;59(1):139–50. doi: 10.1007/s00125-015-3784-4 (PMC4670456; doi:10.1007/s00125-015-3784-4)
Supplement: Supplementary file 8 — (PDF 81 kb) [file 125_2015_3784_MOESM8_ESM.pdf]

ESM Table 1 List of regulated genes by functional category appearing in the heat maps of the main figures

FULL GENE NAME	GENE SYMBOL	WT P1	NULL P1	FOLD	Padj	WT WK8	MUT WK8	FOLD	Padj
**TRANSCRIPTION FACTORS**									
GATA binding protein 4	Gata4	103.70	480.05	4.63	0.03	843.26	134.42	0.16	0.00
insulinoma-associated 1	Insm1	3011.99	1569.74	0.52	0.04	1610.22	3726.25	2.31	0.00
ISL1 transcription factor, LIM/homeodomain	Isl1	1519.68	868.43	0.57	0.00	1125.45	1698.15	1.51	0.43
v-maf musculoaponeurotic fibrosarcoma oncogene family, protein A	Mafa	367.82	178.77	0.49	0.00	543.21	1579.28	2.91	0.00
v-maf musculoaponeurotic fibrosarcoma oncogene family, protein B	Mafb	2276.07	1315.86	0.58	0.01	766.46	664.41	0.87	0.48
motor neuron and pancreas homeobox 1	Mnx1	376.14	130.16	0.35	0.08	134.63	274.07	2.04	0.10
myelin transcription factor 1	Myt1	1268.86	738.70	0.58	0.14	749.03	1411.09	1.88	0.00
neurogenic differentiation 1	Neurod1	2189.51	1238.28	0.57	0.14	1165.05	2489.82	2.14	0.00
nuclear factor of activated T-cells, cytoplasmic, calcineurin-dependent 1	Nfatc1	666.85	940.04	1.41	0.31	603.36	358.79	0.59	0.00
nuclear factor, erythroid derived 2, like 2	Nfe2l2	2015.71	2064.83	1.02	0.99	1791.06	1051.06	0.59	0.00
NK2 transcription factor related, locus 2	Nkx2-2	1669.40	873.31	0.52	0.00	967.18	1610.89	1.67	0.00
NK6 homeobox 1	Nkx6-1	2786.42	1664.19	0.60	0.10	2771.10	5584.75	2.02	0.00
paired box gene 6	Pax6	3267.28	2007.80	0.61	0.02	2208.14	3944.29	1.79	0.01
pancreatic and duodenal homeobox 1	Pdx1	1432.91	819.09	0.57	0.32	1106.58	1907.39	1.72	0.00
**GLYCOLYSIS AND GLUTAMINOLYSIS**									
myelocytomatosis oncogene	Myc	1978.44	2518.44	1.27	0.49	2007.72	510.32	0.25	0.00
hexokinase 2	Hk2	149.66	353.35	2.36	0.09	371.52	48.82	0.13	0.00
hexokinase domain containing 1	Hkdc1	47.75	204.50	4.28	0.00	137.71	30.71	0.22	0.00
enolase 2, gamma neuronal	Eno2	429.52	494.51	1.15	0.79	625.34	1042.20	1.67	0.01
hexokinase 1	Hk1	399.56	571.22	1.43	0.32	514.74	236.86	0.46	0.00
lactate dehydrogenase A	Ldha	1709.74	2420.81	1.42	0.51	1275.18	333.61	0.26	0.00
O-linked N-acetylglucosamine (GlcNAc) transferase	Ogt	1086.16	1804.71	1.66	0.04	1818.34	1366.56	0.75	0.09
6-phosphofructo-2-kinase/fructose-2,6-biphosphatase 2	Pfkfb2	1241.43	852.71	0.69	0.26	2008.54	3648.90	1.82	0.00
pyruvate kinase liver and red blood cell	Pklr	149.73	154.74	1.03	1.00	133.42	218.33	1.64	0.04
glutaminase 2 (liver, mitochondrial)	Gls2	155.30	1009.46	6.50	0.01	1456.42	371.96	0.26	0.00
solute carrier family 2 (facilitated glucose transporter), member 2	Slc2a2	1760.88	819.98	0.47	0.05	2451.73	5046.61	2.06	0.07
glucagon-like peptide 1 receptor	Glp1r	233.49	183.19	0.78	0.77	968.53	1825.27	1.88	0.00
glutamic pyruvic transaminase, soluble	Gpt	107.16	125.84	1.17	0.82	591.21	192.21	0.33	0.00
glutamic pyruvate transaminase (alanine aminotransferase) 2	Gpt2	455.33	606.20	1.33	0.46	1378.41	653.81	0.47	0.00
solute carrier family 2 (facilitated glucose transporter), member 1	Slc2a1	1910.92	2218.12	1.16	0.74	1769.27	716.40	0.40	0.00
solute carrier family 2 (facilitated glucose transporter), member 3	Slc2a3	315.18	429.75	1.36	0.48	130.34	76.24	0.58	0.02
**GLP1R AND cAMP SIGNALLING**									
adenylate cyclase 4	Adcy4	423.63	569.43	1.34	0.46	431.44	155.71	0.36	0.00
adenylate cyclase 5	Adcy5	190.02	218.70	1.15	0.86	144.93	73.38	0.51	0.00
glucagon-like peptide 1 receptor	Glp1r	233.49	183.19	0.78	0.77	968.53	1825.27	1.88	0.00
phosphodiesterase 10A	Pde10a	210.32	217.62	1.03	0.99	291.32	877.72	3.01	0.00
phosphodiesterase 1C	Pde1c	460.11	320.88	0.70	0.67	260.23	470.34	1.81	0.00
protein kinase, cAMP dependent, catalytic, beta	Prkacb	3242.47	2531.31	0.78	0.49	4647.11	7568.29	1.63	0.01
protein kinase, cAMP dependent regulatory, type I beta	Prkar1b	1488.34	1343.43	0.90	0.87	1495.66	2904.30	1.94	0.00

protein kinase, cAMP dependent regulatory, type II beta	Prkar2b	450.05	391.28	0.87	0.79	358.88	580.10	1.62	0.04
Rap guanine nucleotide exchange factor (GEF) 3	Rapgef3	223.53	268.16	1.20	0.76	300.86	160.13	0.53	0.02
Rap guanine nucleotide exchange factor (GEF) 4	Rapgef4	814.02	641.79	0.79	0.48	1067.09	1893.49	1.77	0.00
**OXIDATIVE DAMAGE CONTROL**									
catalase	Cat	644.09	738.30	1.15	0.87	858.18	243.80	0.28	0.00
cytoglobin	Cygb	274.61	338.34	1.23	0.80	362.19	178.78	0.49	0.00
glutaredoxin	Glrx	235.99	173.51	0.74	0.41	171.55	297.70	1.74	0.01
glutathione peroxidase 8	Gpx8	193.88	231.83	1.20	0.84	94.13	25.16	0.27	0.00
glutathione S-transferase, alpha 4	Gsta4	168.64	176.54	1.05	0.99	158.01	18.69	0.12	0.04
glutathione S-transferase kappa 1	Gstk1	52.71	87.60	1.66	0.25	249.87	88.52	0.35	0.00
glutathione S-transferase, mu 1	Gstm1	683.00	909.94	1.33	0.42	1395.09	690.17	0.49	0.00
glutathione S-transferase, mu 2	Gstm2	198.78	225.71	1.14	0.90	921.85	248.49	0.27	0.00
glutathione S-transferase, mu 7	Gstm7	177.71	231.78	1.30	0.65	170.78	62.58	0.37	0.00
glutathione S-transferase, pi 1	Gstp1	481.25	584.92	1.22	0.82	1684.32	464.02	0.28	0.00
glutathione S-transferase, theta 1	Gstt1	259.96	265.45	1.02	1.00	544.92	286.42	0.53	0.00
glutathione S-transferase, theta 2	Gstt2	167.86	214.47	1.28	0.70	309.19	173.24	0.56	0.04
glutathione S-transferase, theta 3	Gstt3	84.79	103.39	1.22	0.84	315.09	65.43	0.21	0.00
microsomal glutathione S-transferase 1	Mgst1	706.87	529.74	0.75	0.44	263.17	53.03	0.20	0.00
microsomal glutathione S-transferase 3	Mgst3	305.71	275.01	0.90	0.91	290.02	158.48	0.55	0.00
methionine sulfoxide reductase A	Msra	122.02	149.40	1.22	0.74	281.32	145.46	0.52	0.00
methionine sulfoxide reductase B3	Msrb3	432.98	616.07	1.42	0.36	270.65	104.54	0.39	0.00
peroxiredoxin 4	Prdx4	830.57	1258.91	1.52	0.60	3971.03	968.36	0.24	0.00
pyrroline-5-carboxylate reductase 1	Pycr1	186.94	440.98	2.36	0.04	1305.04	541.20	0.41	0.00
quiescin Q6 sulfhydryl oxidase 1	Qsox1	1814.71	2165.70	1.19	0.67	3474.48	1835.35	0.53	0.00
selenoprotein X 1	Sepx1	292.32	349.96	1.20	0.74	1600.57	487.43	0.30	0.00
SH3 domain binding glutamic acid-rich protein-like 3	Sh3bgrl3	678.93	548.24	0.81	0.65	481.91	210.68	0.44	0.00
superoxide dismutase 3	Sod3	173.46	193.69	1.12	0.93	125.01	53.72	0.43	0.06
thioredoxin 1	Txn1	2896.52	2456.31	0.85	0.74	2330.22	981.45	0.42	0.00
thioredoxin reductase 1	Txnrd1	4264.44	6824.20	1.60	0.24	7862.32	4504.26	0.57	0.03
thioredoxin reductase 3	Txnrd3	239.49	294.75	1.23	0.71	249.30	136.01	0.55	0.01
**K+ CHANNELS**									
hyperpolarization-activated, cyclic nucleotide-gated K+ 3	Hcn3	226.60	175.44	0.77	0.58	242.90	522.88	2.15	0.00
hyperpolarization-activated, cyclic nucleotide-gated K+ 4	Hcn4	148.06	135.73	0.92	0.95	147.40	329.78	2.24	0.00
potassium voltage-gated channel, shaker-related subfamily, beta member 3	Kcnab3	102.34	75.10	0.73	0.74	151.68	249.15	1.64	0.04
potassium voltage gated channel, Shab-related subfamily, member 2	Kcnb2	50.13	48.54	0.97	0.99	46.74	101.87	2.18	0.01
potassium voltage-gated channel, subfamily H, member 1	Kcnh1	53.93	67.14	1.25	0.83	175.65	450.81	2.57	0.00
similar to potassium channel erg1a; potassium voltage-gated channel, subfamily H, member 2	Kcnh2	1271.17	1010.53	0.79	0.60	856.64	1455.65	1.70	0.00
potassium voltage-gated channel, subfamily H, member 6	Kcnh6	612.02	459.67	0.75	0.42	623.43	1088.96	1.75	0.05
Kv channel-interacting protein 1	Kcnip1	155.91	70.59	0.45	0.00	472.27	1078.33	2.28	0.00
potassium inwardly rectifying channel, subfamily J, member 11	Kcnj11	1484.57	862.48	0.58	0.15	1176.74	2193.99	1.86	0.00
potassium inwardly-rectifying channel, subfamily J, member 12	Kcnj12	40.95	38.40	0.94	0.98	74.54	132.08	1.77	0.05
potassium inwardly-rectifying channel, subfamily J, member 6	Kcnj6	27.55	18.07	0.66	0.64	66.79	150.28	2.25	0.01
potassium channel, subfamily K, member 12	Kcnk12	118.96	92.42	0.78	0.72	206.87	421.18	2.04	0.00
potassium channel, subfamily K, member 16	Kcnk16	1047.64	767.25	0.73	0.30	961.82	1810.23	1.88	0.00
potassium large conductance calcium-activated channel, subfamily M, alpha member 1	Kcnma1	313.22	292.93	0.94	0.98	317.25	779.28	2.46	0.00
potassium large conductance calcium-activated channel, subfamily M, beta member 2	Kcnmb2	671.87	463.36	0.69	0.63	724.60	1241.41	1.71	0.01

potassium intermediate/small conductance calcium-activated channel, subfamily N, member 3	Kcnn3	33.53	44.53	1.33	0.74	176.51	325.11	1.84	0.06
potassium channel tetramerisation domain containing 18	Kctd18	286.56	300.84	1.05	0.98	337.17	698.60	2.07	0.00
potassium channel tetramerisation domain containing 20	Kctd20	2749.00	2307.58	0.84	0.71	2675.31	4563.21	1.71	0.00
potassium channel tetramerisation domain containing 8	Kctd8	69.11	43.02	0.62	0.51	44.79	122.61	2.74	0.00
**Ca2+ CHANNELS**									
calcium channel, voltage-dependent, L type, alpha 1C subunit	Cacna1c	347.03	396.41	1.14	0.85	496.10	953.01	1.92	0.00
calcium channel, voltage-dependent, L type, alpha 1D subunit	Cacna1d	1135.16	1033.35	0.91	0.97	1270.07	2159.31	1.70	0.00
calcium channel, voltage-dependent, beta 2 subunit	Cacnb2	332.73	235.83	0.71	0.54	390.87	732.62	1.87	0.00
transient receptor potential cation channel, subfamily C, member 1	Trpc1	427.25	458.20	1.07	0.97	482.32	786.61	1.63	0.00
transient receptor potential cation channel, subfamily C, member 4	Trpc4	29.37	30.88	1.05	0.99	55.70	102.80	1.85	0.04
**EXOCYTOSIS AND REGULATION**									
adenosine A2b receptor	Adora2b	113.91	47.31	0.42	0.00	107.03	454.89	4.25	0.00
adenosine A3 receptor	Adora3	28.15	16.03	0.57	0.52	124.28	203.31	1.64	0.06
Ca2+-dependent secretion activator	Cadps	955.71	973.65	1.02	1.00	1197.44	1979.18	1.65	0.01
complexin 2	Cplx2	4509.18	3264.98	0.72	0.41	2961.47	4939.92	1.67	0.01
cortactin binding protein 2	Cttnbp2	94.35	139.66	1.48	0.34	291.72	652.19	2.24	0.00
glucagon receptor	Gcgr	523.92	244.91	0.47	0.14	319.01	569.92	1.79	0.00
glycine receptor, beta subunit	Glrb	27.78	31.82	1.15	0.92	264.13	465.59	1.76	0.02
golgi autoantigen, golgin subfamily a, 7B	Gnpda1	150.93	156.19	1.03	0.99	258.20	496.28	1.92	0.00
myosin VIIA and Rab interacting protein	Myrip	96.16	194.58	2.02	0.03	218.72	116.38	0.53	0.01
piccolo (presynaptic cytomatrix protein)	Pclo	816.55	698.07	0.85	0.93	1080.70	2056.36	1.90	0.00
RAB26, member RAS oncogene family	Rab26	49.78	150.68	3.03	0.01	443.05	111.80	0.25	0.00
RAB27A, member RAS oncogene family	Rab27a	2323.10	1392.91	0.60	0.36	2071.42	3456.28	1.67	0.00
RAB3B, member RAS oncogene family	Rab3b	534.11	450.48	0.84	0.88	281.78	498.81	1.77	0.00
Rap guanine nucleotide exchange factor (GEF) 4	Rapgef4	814.02	641.79	0.79	0.48	1067.09	1893.49	1.77	0.00
regulating synaptic membrane exocytosis 2	Rims2	387.80	395.06	1.02	1.00	434.40	810.23	1.87	0.00
secretory carrier membrane protein 5	Scamp5	614.92	494.36	0.80	0.56	500.92	869.85	1.74	0.00
Sec1 family domain containing 1	Scfd1	1316.50	1265.05	0.96	0.98	2334.38	3724.89	1.60	0.00
secernin 1	Scrn1	587.81	479.45	0.82	0.59	698.96	1193.19	1.71	0.02
synaptosomal-associated protein 25	Snap25	1459.75	1316.30	0.90	0.85	1272.61	2288.34	1.80	0.00
six transmembrane epithelial antigen of prostate 2	Steap2	271.13	265.73	0.98	1.00	359.56	657.39	1.83	0.01
syntaxin binding protein 1	Stxbp1	2285.93	1937.72	0.85	0.68	2535.29	4318.75	1.70	0.00
syntaxin binding protein 5-like	Stxbp5l	262.20	241.47	0.92	0.94	239.34	516.45	2.16	0.00
synaptophysin	Syp	2412.60	1660.41	0.69	0.13	2299.96	4483.25	1.95	0.00
synaptotagmin IV	Syt4	315.09	348.90	1.11	0.86	1387.84	3956.53	2.85	0.00
synaptotagmin-like 4	Sytl4	3331.31	1451.69	0.44	0.18	2702.49	5901.24	2.18	0.00
**SECRETORY GRANULE**									
actinin, alpha 1	Actn1	2938.73	5699.28	1.94	0.02	4079.97	1749.31	0.43	0.00
CUB and zona pellucida-like domains 1	Cuzd1	402.35	2717.35	6.75	0.00	25545.73	5293.98	0.21	0.00
deleted in malignant brain tumors 1	Dmbt1	4098.16	20393.29	4.98	0.17	120399.88	28998.81	0.24	0.00
heat shock protein 1	Hspd1	4935.62	7727.60	1.57	0.07	8897.40	12100.40	1.36	0.15
insulin I	Ins1	257147.71	102466.10	0.40	0.07	307185.00	595075.56	1.94	0.00
insulin II	Ins2	405780.89	148466.18	0.37	0.08	764479.22	1559674.83	2.04	0.00
proprotein convertase subtilisin/kexin type 1	Pcsk1	3373.35	1622.84	0.48	0.12	2685.91	5645.04	2.10	0.00

phosphoinositide kinase, FYVE finger containing	Pikfyve	276.50	429.87	1.55	0.25	434.30	752.47	1.73	0.00
RAB37, member of RAS oncogene family	Rab37	1124.05	619.08	0.55	0.00	914.47	1606.56	1.76	0.00
Rac GTPase-activating protein 1	Racgap1	601.26	508.95	0.85	0.77	122.10	232.27	1.90	0.00
rabphilin 3A-like	Rph3al	2141.03	1369.35	0.64	0.04	1600.21	2330.37	1.46	0.04
secretogranin III	Scg3	10606.08	5252.79	0.50	0.22	10386.89	19149.90	1.84	0.00
secretogranin V	Scg5	2935.02	1855.81	0.63	0.03	4919.67	8112.66	1.65	0.01
selectin, platelet	Selp	1147.65	3576.18	3.12	0.00	4018.85	48.34	0.01	0.05
transmembrane domain trafficking protein 2	Tmed2	678.84	491.54	0.72	0.59	927.77	358.96	0.39	0.00
