# Supplementary material for: Aldehyde dehydrogenase activity is necessary for beta cell development and functionality in mice
Source: Diabetologia. 2015 Oct 31;59(1):139–50. doi: 10.1007/s00125-015-3784-4 (PMC4670456; doi:10.1007/s00125-015-3784-4)
Supplement: Supplementary file 9 — (PDF 92 kb) [file 125_2015_3784_MOESM9_ESM.pdf]

ESM Table 2 List of regulated genes by functional category appearing in the heat maps of ESM Figure 5

FULL GENE NAME	GENE SYMBOL	WT P1	NULL P1	FOLD	Padj	WT WK8	MUT WK8	FOLD	Padj
**CELL CYCLE ASSOCIATED GENES**									
adenylate cyclase 3	Adcy3	168.71	214.57	1.27	0.67	146.77	247.37	1.69	0.07
ankyrin repeat domain 54	Ankrd54	1148.00	742.46	0.65	0.06	804.25	1325.93	1.65	0.00
ataxia telangiectasia mutated homolog	Atm	365.14	336.95	0.92	0.92	415.42	664.83	1.60	0.06
cyclin-dependent kinase inhibitor 1A (p21)	Cdkn1a	4622.79	5057.97	1.09	0.87	4339.83	2019.71	0.47	0.00
cyclin-dependent kinase inhibitor 2B (p15)	Cdkn2b	130.27	95.16	0.73	0.55	161.32	264.16	1.64	0.03
centrosomal protein 120	Cep120	865.11	934.70	1.08	0.90	947.06	1641.32	1.73	0.00
centrobin, centrosomal BRCA2 interacting protein	Cntrob	319.50	262.63	0.82	0.67	277.31	462.75	1.67	0.01
cytoplasmic polyadenylation element binding protein 1	Cpeb1	282.22	265.62	0.94	0.97	773.47	1522.79	1.97	0.00
dystonin	Dst	2553.99	2811.22	1.10	0.89	3518.55	5697.57	1.62	0.00
formin 2	Fmn2	1291.41	699.67	0.54	0.52	1213.62	2034.36	1.68	0.00
forkhead box C1	Foxc1	138.34	143.88	1.04	1.00	106.34	34.34	0.32	0.00
growth arrest specific 2	Gas2	270.46	243.15	0.90	0.89	178.69	400.23	2.24	0.00
MAD2L1 binding protein	Mad2l1bp	140.20	128.40	0.92	0.93	191.67	96.20	0.50	0.00
meiosis-specific nuclear structural protein 1	Mns1	168.53	121.49	0.72	0.37	278.52	747.79	2.68	0.00
myosin XVI	Myo16	19.97	90.33	4.52	0.00	209.41	52.90	0.25	0.00
polo-like kinase 3	Plk3	1135.51	884.67	0.78	0.59	1615.27	2844.61	1.76	0.01
Ras association domain family member 4	Rassf4	267.67	233.76	0.87	0.92	207.85	410.32	1.97	0.00
regulator of chromosome condensation (RCC1) and BTB (POZ) domain containing pr	Rcbtb1	750.48	645.54	0.86	0.80	904.74	1553.58	1.72	0.00
REC8 homolog	Rec8	145.21	81.47	0.56	0.04	267.30	486.45	1.82	0.10
ras homolog gene family, member B	Rhob	5554.69	5428.88	0.98	0.99	5037.73	2738.80	0.54	0.00
replication protein A1	Rpa1	832.11	793.51	0.95	0.96	746.30	1205.29	1.62	0.00
radial spoke head 1 homolog	Rsph1	92.12	39.26	0.43	0.09	52.65	102.44	1.95	0.07
sestrin 3	Sesn3	634.04	533.87	0.84	0.71	423.12	922.43	2.18	0.00
TSPY-like 2	Tspyl2	1143.19	1017.52	0.89	0.82	1475.26	2486.79	1.69	0.00
**MITOSIS ASSOCIATED GENES**									
cyclin G2	Ccng2	893.53	772.96	0.87	0.75	520.14	999.47	1.92	0.01
centrosomal protein 192	Cep192	271.59	377.92	1.39	0.48	240.33	386.05	1.61	0.02
checkpoint kinase 1 homolog	Chek1	137.28	258.26	1.88	0.23	134.22	253.83	1.89	0.03
cytoskeleton associated protein 5	Ckap5	1192.54	1130.48	0.95	0.95	789.74	1300.85	1.65	0.00
CLIP associating protein 2	Clasp2	1003.78	1219.46	1.21	0.61	1086.25	1824.08	1.68	0.00
microtubule-associated protein 9	Mtap9	489.29	404.79	0.83	0.67	407.26	692.08	1.70	0.00
nuclear distribution gene C homolog	Nudc	1751.90	1438.88	0.82	0.61	2199.90	3616.36	1.64	0.00
protein phosphatase 2, regulatory subunit B	Ppp2r3a	679.89	651.76	0.96	0.99	556.33	1063.94	1.91	0.00
ribosomal protein S6 kinase, polypeptide 2	Rps6ka2	849.77	751.01	0.88	0.85	797.54	1589.04	1.99	0.00
SPC25, NDC80 kinetochore complex component	Spc25	701.99	372.76	0.53	0.11	1852.02	3833.02	2.07	0.00
**K+ AND Ca2+ TRANSPORT**									
ATPase, Ca++ transporting, cardiac muscle, slow twitch 2	Atp2a2	10004.90	9441.32	0.94	0.93	14333.98	25427.53	1.77	0.00
ATPase, Ca++ transporting, ubiquitous	Atp2a3	4171.28	2214.10	0.53	0.05	6450.54	11008.38	1.71	0.08
ATPase, Ca++ transporting, plasma membrane 2	Atp2b2	131.90	112.75	0.85	0.81	354.89	626.84	1.77	0.02

ATPase, H+/K+ exchanging, gastric, alpha polypeptide	Atp4a	144.38	75.63	0.52	0.05	63.48	139.77	2.20	0.03
solute carrier family 12, member 7	Slc12a7	995.76	882.09	0.89	0.88	1095.83	1931.18	1.76	0.00
solute carrier family 24 (sodium/potassium/calcium exchanger), member 2	Slc24a2	330.34	261.50	0.79	0.79	919.87	1745.96	1.90	0.01
solute carrier family 24 (sodium/potassium/calcium exchanger), member 3	Slc24a3	45.73	77.79	1.70	0.53	65.48	26.83	0.41	0.02
solute carrier family 24 (sodium/potassium/calcium exchanger), member 6	Slc24a6	148.04	130.23	0.88	0.88	238.20	106.20	0.45	0.01
solute carrier family 8 (sodium/calcium exchanger), member 1	Slc8a1	246.83	339.41	1.38	0.55	190.95	379.41	1.99	0.00

CYTOPLASMIC VESICLE

ALS2 C-terminal like	Als2cl	416.49	687.82	1.65	0.08	644.63	216.67	0.34	0.00
amphiphysin	Amph	125.96	160.81	1.28	0.66	275.80	505.16	1.83	0.00
adaptor-related protein complex 1, sigma 2 subunit	Ap1s2	2100.76	1611.76	0.77	0.56	2344.67	3926.50	1.67	0.00
adaptor-related protein complex 3, beta 2 subunit	Ap3b2	954.07	705.70	0.74	0.29	1117.71	2217.80	1.98	0.00
ataxia telangiectasia mutated homolog (human)	Atm	365.14	336.95	0.92	0.92	415.42	664.83	1.60	0.06
ATPase, Cu++ transporting, alpha polypeptide	Atp7a	482.95	582.24	1.21	0.88	481.22	1006.43	2.09	0.00
ATPase, aminophospholipid transporter (APLT), class I, type 8A, member 1	Atp8a1	292.98	435.35	1.49	0.26	483.75	826.64	1.71	0.00
coiled coil domain containing 88A	Ccdc88a	596.02	713.18	1.20	0.72	657.59	1073.27	1.63	0.00
cystic fibrosis transmembrane conductance regulator homolog	Cftr	54.18	145.32	2.68	0.01	132.02	20.33	0.15	0.00
cysteine-rich hydrophobic domain 1	Chic1	1457.31	875.98	0.60	0.20	2270.46	4825.41	2.13	0.00
disabled homolog 2 (Drosophila)	Dab2	365.42	647.84	1.77	0.04	416.60	200.95	0.48	0.13
dystonin; hypothetical protein LOC100047109	Dst	2553.99	2811.22	1.10	0.89	3518.55	5697.57	1.62	0.00
epidermal growth factor receptor	Egfr	137.36	348.46	2.54	0.04	563.12	489.81	0.87	0.70
gamma-glutamyl hydrolase	Ggh	365.57	814.69	2.23	0.05	4827.37	1084.02	0.22	0.00
glutamate receptor, ionotropic, NMDA1 (zeta 1)	Grin1	282.39	212.99	0.75	0.63	386.77	728.57	1.88	0.00
glutamate receptor interacting protein 1	Grip1	250.35	181.97	0.73	0.56	175.35	335.00	1.91	0.00
huntingtin-associated protein 1	Hap1	3421.98	2607.33	0.76	0.35	2732.42	4436.67	1.62	0.00
myosin, heavy polypeptide 11, smooth muscle	Myh11	261.96	599.96	2.29	0.00	864.02	378.48	0.44	0.00
myosin VIIA and Rab interacting protein	Myrip	96.16	194.58	2.02	0.03	218.72	116.38	0.53	0.01
secretagogin, EF-hand calcium binding protein	Scgn	3188.74	1811.28	0.57	0.05	2153.86	3116.62	1.45	0.05
serum/glucocorticoid regulated kinase 3	Sgk3	308.76	247.03	0.80	0.79	211.27	353.41	1.67	0.02
SH3-domain kinase binding protein 1	Sh3kbp1	330.31	289.19	0.88	0.83	318.04	530.70	1.67	0.01
solute carrier family 30 (zinc transporter), member 8	Slc30a8	3733.76	2459.37	0.66	0.44	5563.65	10454.67	1.88	0.00
synaptic vesicle glycoprotein 2 a	Sv2a	686.50	513.47	0.75	0.34	758.16	1341.49	1.77	0.01
syncollin	Sycn	3420.37	16133.12	4.72	0.04	143907.40	27347.08	0.19	0.00
synapsin I	Syn1	398.32	371.97	0.93	0.96	471.65	901.43	1.91	0.00
synaptoporin	Synpr	153.69	96.21	0.63	0.14	161.97	368.41	2.27	0.00
synaptotagmin XII	Syt12	53.58	119.91	2.24	0.07	54.59	20.29	0.37	0.01
synaptotagmin XIII	Syt13	7508.77	4043.95	0.54	0.16	6353.84	12710.86	2.00	0.00
synaptotagmin V	Syt5	399.79	245.00	0.61	0.05	311.87	534.09	1.71	0.01
synaptotagmin VII	Syt7	2972.81	2095.14	0.70	0.59	2318.69	4142.44	1.79	0.00
tyrosine hydroxylase	Th	203.57	208.87	1.03	1.00	317.30	574.06	1.81	0.07

GTPase REGULATORY ACTIVITY

ArfGAP with dual PH domains 2	Adap2	83.38	112.68	1.35	0.72	344.51	115.31	0.33	0.00
A kinase (PRKA) anchor protein 13	Akap13	1231.81	1916.57	1.56	0.14	2052.46	3398.81	1.66	0.00
ALS2 C-terminal like	Als2cl	416.49	687.82	1.65	0.08	644.63	216.67	0.34	0.00
ArfGAP with RhoGAP domain, ankyrin repeat and PH domain 2	Arap2	249.90	451.23	1.81	0.05	391.01	84.76	0.22	0.00
ArfGAP with RhoGAP domain, ankyrin repeat and PH domain 3	Arap3	905.55	1075.80	1.19	0.71	676.47	383.53	0.57	0.00
Rho GTPase activating protein 10	Arhgap10	224.71	288.90	1.29	0.65	263.40	86.19	0.33	0.00

Rho GTPase activating protein 11A	Arhgap11a	360.18	337.63	0.94	0.95	67.28	118.11	1.76	0.03
Rho GTPase activating protein 27	Arhgap27	340.95	373.62	1.10	0.93	284.25	107.60	0.38	0.00
Rho GTPase activating protein 28	Arhgap28	328.74	601.72	1.83	0.15	268.85	47.74	0.18	0.00
CDC42 GTPase-activating protein	Arhgap31	673.02	851.31	1.26	0.65	600.93	340.53	0.57	0.00
Rho GDP dissociation inhibitor (GDI) gamma	Arhgdig	2885.81	10034.80	3.48	0.09	51286.39	9267.64	0.18	0.00
Rho guanine nucleotide exchange factor (GEF) 10-like	Arhgef10l	470.35	630.65	1.34	0.45	566.47	253.21	0.45	0.00
Rho guanine nucleotide exchange factor (GEF) 15	Arhgef15	342.54	361.55	1.06	0.98	231.30	99.47	0.43	0.00
Rho guanine nucleotide exchange factor (GEF) 19	Arhgef19	189.84	215.84	1.14	0.89	339.95	171.56	0.50	0.01
ArfGAP with SH3 domain, ankyrin repeat and PH domain 3	Asap3	110.88	220.34	1.99	0.04	208.45	87.73	0.42	0.09
similar to choroidermia	Chm	426.14	442.65	1.04	0.99	447.53	718.70	1.61	0.01
cytohesin 3	Cyth3	1369.01	1559.72	1.14	0.82	806.54	467.14	0.58	0.00
deleted in liver cancer 1	Dlc1	1165.73	1647.67	1.41	0.32	1206.91	619.68	0.51	0.00
dedicator of cytokinesis 1	Dock1	949.03	1383.70	1.46	0.31	748.58	372.59	0.50	0.01
dedicator of cytokinesis 10	Dock10	221.68	376.18	1.70	0.33	306.61	560.72	1.83	0.00
dedicator of cytokinesis 4	Dock4	197.55	421.37	2.13	0.04	195.16	114.80	0.59	0.03
dedicator of cytokinesis 5	Dock5	190.21	381.04	2.00	0.18	440.82	217.29	0.49	0.00
ect2 oncogene	Ect2	285.17	276.46	0.97	0.99	58.72	149.89	2.55	0.00
exophilin 5	Exph5	211.14	210.88	1.00	1.00	462.23	844.14	1.83	0.00
FYVE, RhoGEF and PH domain containing 3	Fgd3	1705.16	2523.29	1.48	0.51	918.56	112.55	0.12	0.00
FYVE, RhoGEF and PH domain containing 5	Fgd5	439.06	534.36	1.22	0.73	306.74	157.98	0.52	0.01
GH regulated TBC protein 1	Grtp1	312.39	202.24	0.65	0.13	285.53	496.23	1.74	0.01
IQ motif containing GTPase activating protein 2	Iqgap2	339.14	510.94	1.51	0.30	635.88	256.55	0.40	0.00
IQ motif and Sec7 domain 1	Iqsec1	602.23	773.03	1.28	0.62	485.93	225.49	0.46	0.00
MAP-kinase activating death domain	Madd	1544.51	1105.23	0.72	0.19	2184.89	3913.41	1.79	0.00
myosin VIIA and Rab interacting protein	Myrip	96.16	194.58	2.02	0.03	218.72	116.38	0.53	0.01
neuroepithelial cell transforming gene 1	Net1	473.51	606.19	1.28	0.56	513.43	225.95	0.44	0.00
neuronal guanine nucleotide exchange factor	Ngef	249.18	281.23	1.13	0.87	183.43	62.99	0.34	0.00
phospholipase C, epsilon 1	Plce1	289.17	502.43	1.74	0.14	285.27	131.68	0.46	0.00
pleckstrin homology domain containing, family G (with RhoGef domain) member 2	Plekhg2	999.69	1274.09	1.27	0.56	518.35	203.95	0.39	0.00
Rho GTPase activating protein 8	Prr5	194.55	237.48	1.22	0.77	145.45	78.42	0.54	0.02
rabaptin, RAB GTPase binding effector protein 1	Rabep1	1140.65	1221.85	1.07	0.92	1148.33	1872.42	1.63	0.00
Rac GTPase-activating protein 1	Racgap1	601.26	508.95	0.85	0.77	122.10	232.27	1.90	0.00
Rap1 GTPase-activating protein	Rap1gap	1982.95	4663.71	2.35	0.06	7269.52	2126.48	0.29	0.00
Rap guanine nucleotide exchange factor (GEF) 3	Rapgef3	223.53	268.16	1.20	0.76	300.86	160.13	0.53	0.02
Rap guanine nucleotide exchange factor (GEF) 4	Rapgef4	814.02	641.79	0.79	0.48	1067.09	1893.49	1.77	0.00
Rap guanine nucleotide exchange factor (GEF) 5	Rapgef5	272.47	437.07	1.60	0.27	597.46	215.47	0.36	0.00
RAS p21 protein activator 4	Rasa4	193.61	197.42	1.02	1.00	161.42	65.92	0.41	0.01
RAS protein-specific guanine nucleotide-releasing factor 1	Rasgrf1	335.58	190.72	0.57	0.21	366.38	917.44	2.50	0.00
RAS, guanyl releasing protein 2	Rasgrp2	202.36	208.58	1.03	1.00	154.56	54.94	0.36	0.00
ral guanine nucleotide dissociation stimulator,-like 1	Rgl1	694.46	823.67	1.19	0.71	603.18	982.48	1.63	0.00
regulator of G-protein signaling 17	Rgs17	485.30	368.87	0.76	0.48	165.73	391.68	2.36	0.00
resistance to inhibitors of cholinesterase 8 homolog B	Ric8b	632.75	583.52	0.92	0.90	866.01	1515.45	1.75	0.00
regulating synaptic membrane exocytosis 2	Rims2	387.80	395.06	1.02	1.00	434.40	810.23	1.87	0.00
Ras and Rab interactor 1	Rin1	114.27	237.72	2.08	0.14	207.38	43.64	0.21	0.00
Ras and Rab interactor 3	Rin3	194.32	232.85	1.20	0.81	163.30	79.56	0.49	0.05
retinitis pigmentosa GTPase regulator	Rpgr	296.04	232.79	0.79	0.64	208.55	382.47	1.83	0.01
rhotekin	Rtkn	379.60	450.82	1.19	0.75	435.41	168.53	0.39	0.00
small G protein signaling modulator 1	Sgsm1	1290.88	891.83	0.69	0.19	1718.04	3003.06	1.75	0.00
small G protein signaling modulator 2	Sgsm2	474.65	464.71	0.98	0.99	540.38	915.09	1.69	0.00
SLIT-ROBO Rho GTPase activating protein 1	Srgap1	470.46	636.13	1.35	0.50	147.24	307.29	2.09	0.00

SLIT-ROBO Rho GTPase activating protein 2	Srgap2	584.04	760.82	1.30	0.55	509.73	275.49	0.54	0.00
synapse defective 1, Rho GTPase, homolog 1	Syde1	305.16	352.97	1.16	0.82	226.46	46.96	0.21	0.00
synaptotagmin-like 4	Sytl4	3331.31	1451.69	0.44	0.18	2702.49	5901.24	2.18	0.00
TBC1 domain family, member 24	Tbc1d24	213.46	263.32	1.23	0.66	394.29	655.78	1.66	0.00
TBC1 domain family, member 2B	Tbc1d2b	643.64	847.21	1.32	0.51	385.92	223.72	0.58	0.04
thymus cell antigen 1, theta	Thy1	616.06	645.00	1.05	0.98	316.84	101.17	0.32	0.00
WD repeat domain 67	Wdr67	169.69	236.15	1.39	0.47	146.37	306.12	2.09	0.00
**RIBOSOMAL GENES**									
mitochondrial ribosomal protein L1	Mrpl1	606.88	388.99	0.64	0.08	429.07	667.38	1.56	0.03
mitochondrial ribosomal protein L15	Mrpl15	323.29	326.55	1.01	1.00	605.07	351.72	0.58	0.00
mitochondrial ribosomal protein L52	Mrpl52	494.32	501.99	1.02	1.00	576.15	333.84	0.58	0.00
mitochondrial ribosomal protein S24	Mrps24	393.62	380.44	0.97	0.99	617.43	364.42	0.59	0.00
MRT4, mRNA turnover 4	Mrto4	337.13	405.48	1.20	0.83	298.20	155.48	0.52	0.00
ribosomal protein L10-like	Rpl10	291.49	402.18	1.38	0.42	398.03	164.36	0.41	0.00
ribosomal protein L10A	Rpl10a	508.83	636.62	1.25	0.66	538.47	215.14	0.40	0.00
ribosomal protein L12	Rpl12	314.67	369.13	1.17	0.80	382.98	176.02	0.46	0.00
ribosomal protein L13	Rpl13	662.76	607.34	0.92	0.92	580.32	282.31	0.49	0.00
ribosomal protein L13A	Rpl13a	11141.84	10667.76	0.96	0.96	14259.01	7667.80	0.54	0.00
ribosomal protein L14	Rpl14	2201.90	2061.88	0.94	0.92	1922.06	1023.79	0.53	0.00
ribosomal protein L18	Rpl18	1931.79	1734.08	0.90	0.87	1816.39	1013.95	0.56	0.00
ribosomal protein L18A	Rpl18a	3232.03	3257.35	1.01	1.00	3662.93	1464.15	0.40	0.00
ribosomal protein L19	Rpl19	1170.33	1152.68	0.98	1.00	1209.70	763.76	0.63	0.00
ribosomal protein L21	Rpl21	463.68	452.21	0.98	1.00	512.63	265.29	0.52	0.00
ribosomal protein L22	Rpl22	3432.21	3189.51	0.93	0.91	3137.39	1798.21	0.57	0.00
ribosomal protein L22 like 1	Rpl22l1	1161.84	1070.97	0.92	0.91	1745.87	971.33	0.56	0.00
ribosomal protein L23	Rpl23	3369.63	3385.80	1.00	1.00	3625.60	1770.14	0.49	0.00
ribosomal protein L26	Rpl26	2452.09	2159.32	0.88	0.82	2774.77	1279.10	0.46	0.00
ribosomal protein L27a	Rpl27a	549.57	431.15	0.78	0.57	556.76	338.65	0.61	0.00
ribosomal protein L29	Rpl29	713.97	667.52	0.93	0.93	743.14	319.64	0.43	0.00
ribosomal protein L3	Rpl3	894.67	1040.88	1.16	0.75	1031.44	524.99	0.51	0.00
ribosomal protein L30	Rpl30	2261.42	2196.38	0.97	0.99	2341.70	966.37	0.41	0.00
ribosomal protein L32	Rpl32	6233.83	6409.49	1.03	1.00	9726.23	3709.31	0.38	0.00
ribosomal protein L34	Rpl34	2751.45	2653.63	0.96	0.98	2741.64	1458.22	0.53	0.00
ribosomal protein L35	Rpl35	1971.58	2423.14	1.23	0.76	3137.64	1201.86	0.38	0.00
ribosomal protein L35A	Rpl35a	1929.90	1806.56	0.94	0.93	2006.31	1133.85	0.57	0.00
ribosomal protein L36	Rpl36	197.09	193.76	0.98	1.00	173.20	101.85	0.59	0.03
ribosomal protein L37	Rpl37	2125.75	1989.81	0.94	0.93	2640.34	1293.09	0.49	0.00
ribosomal protein L37A	Rpl37a	3418.91	3076.11	0.90	0.86	2474.56	1467.59	0.59	0.00
ribosomal protein L39	Rpl39	2097.85	1931.09	0.92	0.90	1855.05	978.83	0.53	0.00
ribosomal protein L4	Rpl4	17472.20	19298.47	1.10	0.86	23147.65	13020.52	0.56	0.00
ribosomal protein L41	Rpl41	4216.35	4287.39	1.02	1.00	6779.11	3225.47	0.48	0.00
ribosomal protein L5	Rpl5	429.56	357.68	0.83	0.74	297.84	98.68	0.33	0.00
ribosomal protein L6	Rpl6	2295.32	2371.99	1.03	0.99	2348.82	1291.28	0.55	0.00
ribosomal protein L7	Rpl7	4833.03	5009.74	1.04	0.98	5176.66	2872.62	0.55	0.00
ribosomal protein L7A	Rpl7a	1298.43	1344.30	1.04	0.99	1398.41	804.45	0.58	0.00
ribosomal protein L8	Rpl8	10640.22	10545.54	0.99	1.00	11489.13	6336.69	0.55	0.00
ribosomal protein L9	Rpl9	367.59	339.69	0.92	0.92	303.69	181.01	0.60	0.03
ribosomal protein, large, P0	Rplp0	11662.31	13522.83	1.16	0.75	13020.78	6320.93	0.49	0.00

ribosomal protein, large, P1	Rplp1	8667.85	9583.47	1.11	0.94	15058.10	6275.36	0.42	0.00
ribosomal protein, large P2	Rplp2	5269.12	4829.73	0.92	0.89	4399.49	2107.73	0.48	0.00
ribosomal protein S10	Rps10	2610.10	2212.11	0.85	0.76	2680.52	1181.35	0.44	0.00
ribosomal protein S11	Rps11	5864.45	6403.99	1.09	0.89	5170.77	2462.41	0.48	0.00
ribosomal protein S14	Rps14	8065.63	6880.31	0.85	0.71	8084.34	4478.27	0.55	0.00
ribosomal protein S15	Rps15	5664.41	5350.17	0.94	0.95	6507.83	3296.77	0.51	0.00
ribosomal protein S15A	Rps15a	3141.35	3013.46	0.96	0.98	2402.73	1187.09	0.49	0.00
ribosomal protein S17	Rps17	434.19	475.52	1.10	0.92	572.49	344.45	0.60	0.00
ribosomal protein S18	Rps18	2148.72	2150.28	1.00	1.00	2309.38	970.01	0.42	0.00
ribosomal protein S19	Rps19	2916.79	2626.21	0.90	0.91	2931.82	1282.34	0.44	0.00
ribosomal protein S2	Rps2	3960.29	4235.87	1.07	0.96	4217.60	1870.75	0.44	0.00
ribosomal protein S20	Rps20	2389.22	2203.75	0.92	0.93	2291.33	1109.00	0.48	0.00
ribosomal protein S21	Rps21	4972.68	4527.40	0.91	0.86	5678.47	3300.68	0.58	0.00
ribosomal protein S24	Rps24	3759.04	3638.98	0.97	0.99	5248.55	2264.69	0.43	0.00
ribosomal protein S25	Rps25	1383.09	1430.84	1.03	0.99	1717.11	850.73	0.50	0.00
ribosomal protein S26	Rps26	2599.01	2535.88	0.98	0.99	2384.49	1084.20	0.45	0.00
ribosomal protein S27-like	Rps27l	1113.69	1204.62	1.08	0.98	2490.27	1047.15	0.42	0.00
ribosomal protein S28	Rps28	2626.54	2390.64	0.91	0.88	2575.73	1113.75	0.43	0.00
ribosomal protein S3	Rps3	13759.43	13435.18	0.98	0.99	14484.81	7600.90	0.52	0.00
ribosomal protein S3A	Rps3a	2877.36	2835.04	0.99	1.00	2841.60	1684.39	0.59	0.00
ribosomal protein S4, X-linked	Rps4x	5462.15	6117.85	1.12	0.84	4826.11	2325.52	0.48	0.00
ribosomal protein S5	Rps5	9344.63	9308.65	1.00	1.00	8855.53	4396.37	0.50	0.00
ribosomal protein S6 kinase polypeptide 1	Rps6ka1	361.81	464.69	1.28	0.58	341.06	117.44	0.34	0.00
ribosomal protein S6 kinase, polypeptide 2	Rps6ka2	849.77	751.01	0.88	0.85	797.54	1589.04	1.99	0.00
ribosomal protein S6 kinase-like 1	Rps6kl1	103.83	86.31	0.83	0.77	173.02	348.87	2.02	0.01
ribosomal protein S7	Rps7	190.72	171.43	0.90	0.89	245.40	116.07	0.47	0.00
ribosomal protein S8	Rps8	1280.97	1173.02	0.92	0.94	1619.71	692.49	0.43	0.00
ribosomal protein S9	Rps9	7114.12	7093.41	1.00	1.00	7644.02	3602.60	0.47	0.00
ribosomal protein SA	Rpsa	1442.89	1469.07	1.02	1.00	1675.70	807.49	0.48	0.00
ubiquitin A-52 residue ribosomal protein fusion product 1	Uba52	353.26	379.35	1.07	0.95	532.62	243.58	0.46	0.00
