# Supplementary material for: Aldehyde dehydrogenase activity is necessary for beta cell development and functionality in mice
Source: Diabetologia. 2015 Oct 31;59(1):139–50. doi: 10.1007/s00125-015-3784-4 (PMC4670456; doi:10.1007/s00125-015-3784-4)
Supplement: Supplementary file 10 — (PDF 39 kb) [file 125_2015_3784_MOESM10_ESM.pdf]

ESM Table 3 Expression levels by RNA Seq of *Aldh* genes during pancreas development

GENE SYMBOL	13.5 dpc	14.5 dpc	15.5 dpc
Aldh1l1	68	83	442
Aldh1a1	128	157	810
Aldh4a1	459	359	786
Aldh6a1	562	478	844
Aldh3a2	618	550	706
Aldh1a2	767	733	724
Aldh5a1	917	1025	1050
Aldh16a1	955	768	1230
Aldh9a1	1253	1257	2386
Aldh18a1	2123	2189	2968
Aldh2	2086	1911	2224
Aldh7a1	2402	2345	4099
Aldh1b1	5061	5153	8101
